# Extracellular Adenosine Mediates a Systemic Metabolic Switch during Immune Response

**DOI:** 10.1371/journal.pbio.1002135

**Published:** 2015-04-27

**Authors:** Adam Bajgar, Katerina Kucerova, Lucie Jonatova, Ales Tomcala, Ivana Schneedorferova, Jan Okrouhlik, Tomas Dolezal

**Affiliations:** 1 Faculty of Science, University of South Bohemia in Ceske Budejovice, Ceske Budejovice, Czech Republic; 2 Institute of Parasitology, Biology Centre, Academy of Sciences of the Czech Republic, Ceske Budejovice, Czech Republic; King's College London, UNITED KINGDOM

## Abstract

Immune defense is energetically costly, and thus an effective response requires metabolic adaptation of the organism to reallocate energy from storage, growth, and development towards the immune system. We employ the natural infection of Drosophila with a parasitoid wasp to study energy regulation during immune response. To combat the invasion, the host must produce specialized immune cells (lamellocytes) that destroy the parasitoid egg. We show that a significant portion of nutrients are allocated to differentiating lamellocytes when they would otherwise be used for development. This systemic metabolic switch is mediated by extracellular adenosine released from immune cells. The switch is crucial for an effective immune response. Preventing adenosine transport from immune cells or blocking adenosine receptor precludes the metabolic switch and the deceleration of development, dramatically reducing host resistance. Adenosine thus serves as a signal that the “selfish” immune cells send during infection to secure more energy at the expense of other tissues.

## Abstract 2

A study of the fruit fly's response to parasitoid wasp eggs reveals that immune cells selfishly release adenosine as a signal to trigger a systemic metabolic switch, thereby suppressing nonimmune processes and securing energy and nutrients for immune activity. Read the Primer.

## Author Summary

The immune response is energetically costly and often requires adaption of the whole organism to ensure it receives enough energy. It is not well understood how distribution of energy resources within the organism is regulated during an immune response. To understand this better, we used parasitoid wasp infection of fruit fly larvae—the host larvae have 48 h before they pupate to destroy the infecting “alien” or face destruction by the parasitoid that will consume the developing pupa. Here we find a signal, generated by the host immune cells, which mediates a systemic energy switch. This signal—adenosine—suppresses processes driving larval to pupal development of the host, thereby freeing up energy for the immune system. We show that the resulting developmental delay in the fruit fly larvae is crucial for an efficient immune response; without the adenosine signal, resistance to the parasitoid drops drastically. Generation of this signal by immune cells demonstrates that in response to external stressors, the immune system can mobilize reallocation to itself of energy and nutrients from the rest of the organism.

## Introduction

Immune response is energetically costly [1,2]. Immune cells, upon activation, favor glycolysis over oxidative phosphorylation for fast, albeit inefficient, energy generation and macromolecule synthesis [3,4]. This metabolic shift requires extra glucose as glycolysis produces much less ATP than does oxidative phosphorylation [5]. Therefore, at the organismal level, the energy shifts from storage and nonimmune processes towards the needs of the immune system [6–9].

Regulation of energy during the immune response is critical—full response requires a significant amount of energy, and inability to provide it with nutrients can lead to immune system suppression and reduced resistance [10–12]. In mammalian systems, the inflammatory cytokines TNF-α, IFN-γ, IL-1, and IL-6 are released upon recognition of the pathogen and, besides modulating immune functions, they also stimulate energy release [2,13–16]. Immune cells must respond rapidly to the activating signals, and thus they change their metabolism, which involves, at least in mammalian systems, the preferential use of aerobic glycolysis, known as the Warburg effect [3,4,17]. The increased demand for energy by the immune system requires, both in vertebrates and invertebrates, adaptation of the whole organism, which is associated with an overall metabolic suppression and a systemic insulin resistance in all tissues except the immune cells [2,12,18]. The importance of the systemic regulation of energy is demonstrated by examples of certain infections leading to depletion of energy reserves (wasting) and eventually death of the organism [15,19]. Despite the importance of the systemic regulation of energy, we have only fragmentary knowledge about the molecular mechanisms involved in the regulation of energy during immune response at the organismal level and about the communication between different parts of the organism mediating the shift of energy from storage and growth towards immunity [12,20,21].

Extracellular adenosine (e-Ado) is a signal originating from damaged or stressed tissues. Acting as an energy sensor, e-Ado is released from metabolically stressed cells with depleted ATP [22,23] or made from extracellular ATP leaking from damaged tissues [24]. e-Ado then works as a local or systemic hormone, adjusting metabolism by acting either via adenosine receptors or by the uptake into the cells and conversion to AMP activating AMP-activated protein kinase (AMPK) [24,25]. These actions lead to a suppression of energy consuming processes [22,26–29] and to a release of energy from stores [30].

Damaged tissues and metabolically stressed cells are very likely to occur during immune response and thus it is not surprising that elevated levels of e-Ado are also detected, for example, during sepsis in humans [31]. The capacity of e-Ado to regulate energy metabolism, to “measure” the level of tissue and organismal stress, and to adapt the energy use to the actual situation all make e-Ado a perfect candidate for an energy regulator during immune response. However, the mode of e-Ado action under immune challenge is unclear, as the role of e-Ado in energy regulation has mainly been studied in relation to anoxia in anoxia-tolerant organisms such as turtles and hypoxia and ischemia in rodent models and human patients [22,30], while e-Ado has thus far been associated with mammalian immune response only through its immunomodulatory and anti-inflammatory function [24,32].

We, and others, have previously shown that adenosine regulatory and signaling network in *Drosophila* is similar to mammalian systems [33–37]. In addition, we have shown that e-Ado regulates energy metabolism in *Drosophila*. Increase of e-Ado levels caused by a deficiency of adenosine deaminase-related growth factor A (ADGF-A) leads to hyperglycemia and reduced energy storage [38]. We have also found that the regulation of e-Ado by ADGF-A is particularly important during parasitoid wasp infection in *Drosophila* larvae; ADGF-A is strongly expressed in immune cells that encapsulate the invading wasp egg [39]. These findings further support a potential role of e-Ado in energy regulation during immune response.

Here, we use the parasitoid wasp infection as a model to study the energy regulation during immune reaction. Parasitoid wasps inject their eggs into *Drosophila* larvae, and if the fly larva does not destroy the egg in time, the hatched wasp larva will consume the host [40]. The fly larva recognizes the egg and mounts a robust immune response that involves proliferation and differentiation of specialized immune cells, lamellocytes, which eventually encapsulate the parasitoid egg. Using this immune response as a model, we traced the dietary glucose destinations, measured selected metabolites and gene expressions, and analyzed host resistance and the impact of the immune response on its development.

We describe here the systemic changes in energy metabolism during the immune challenge and the role of e-Ado in the regulation of these changes. We have found that e-Ado, released from the immune cells, mediates a metabolic switch characterized by the suppression of nutrient storage and developmental growth in favor of the immune defense. This metabolic switch—a tradeoff between development and defense—is crucial for the resistance to infection. In *Drosophila* larvae lacking adenosine signaling, development is not suppressed, and the resistance dramatically drops.

## Results

### Immune Response to Parasitoid Wasp Egg

**Fig 1:**
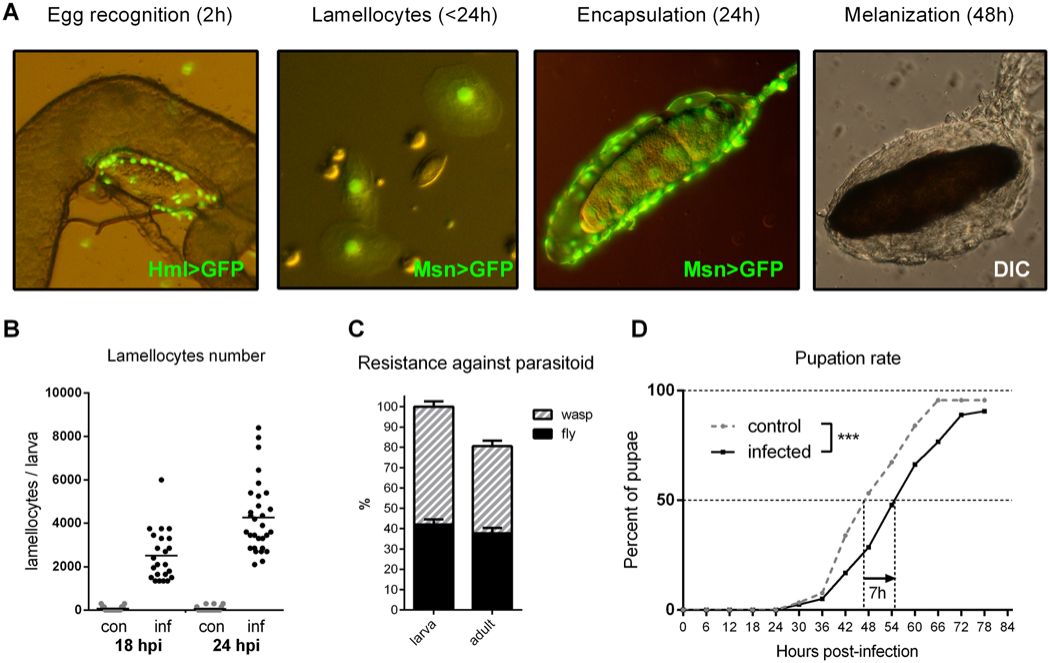
Immune response to parasitoid wasp intrusion. (A) Progressive stages of the response. The wasp egg is recognized by plasmatocytes (green, Hml>GFP) within 2 hpi. Lamellocytes, labeled by the Msn>GFP marker appear in circulation (<24 hpi) and start to encapsulate the egg. At 48 hpi, the egg is fully encapsulated by a multilayer of immune cells and melanized (original image of encapsulation published in [39]). (B) Number of lamellocytes per larva in control (con, grey) and infected (inf, black) larvae at 18 and 24 hpi. Each dot represents an individual larva; the horizontal lines indicate mean. (C) Percentage of host larvae with melanized wasp eggs (black, left column, mean 42%) and surviving host adults (black, right column, mean 38%) against winning wasp larvae and adults (hatched columns). Values are mean ± standard error of measurement (SEM). (D) Pupation of infected larvae (*n* = 316) was significantly delayed compared to control larvae (*n* = 344). Log-rank survival analysis (*p* < 0.0001).

The endoparasitoid wasp *Leptopilina boulardi* injects its egg in early third-instar *Drosophila* larva. The egg, usually hiding in gut folds, is first recognized by the host-circulating hemocytes (Fig 1A) and the recognition triggers immune response [40]. This involves production of specialized cells called lamellocytes (Fig 1A and 1B) within the first 24 h postinfection (hpi; 0 hpi is the time of infection and corresponds to 72 h after egg laying; the time in hpi is also used for the uninfected control). Lamellocytes are then released into circulation, and the egg gets encapsulated with subsequent melanization by 48 hpi (Fig 1A). Production of lamellocytes involves a transient proliferation of prohemocytes in the lymph gland and their terminal differentiation into lamellocytes [41]. The efficiency of egg encapsulation depends on the ability to produce lamellocytes and thus varies among different genetic strains of *Drosophila* [42,43]. Our model was based on the Canton S strain of *Drosophila melanogaster* bearing the *w* ^*1118*^ mutation (hereafter *w*), which served as a control genotype in all our experiments (the term “control” is reserved hereafter for uninfected situations, i.e., control *w* means uninfected *w* larvae). On average, 42% of these *w* host larvae succeeded to destroy the wasp egg and 38% survived to adulthood while 42% parasitoids developed to adult wasps (Fig 1C).

### Immune Response to Parasitoid Egg Invasion Demands Energy

Parasitoid-infected third-instar larvae experienced a 15% developmental delay, pupating on average 7 h later than uninfected controls (Fig 1D). Such a delay might result from redistribution of energy from development towards immune defense. We therefore examined various energy aspects during infection.

**Fig 2:**
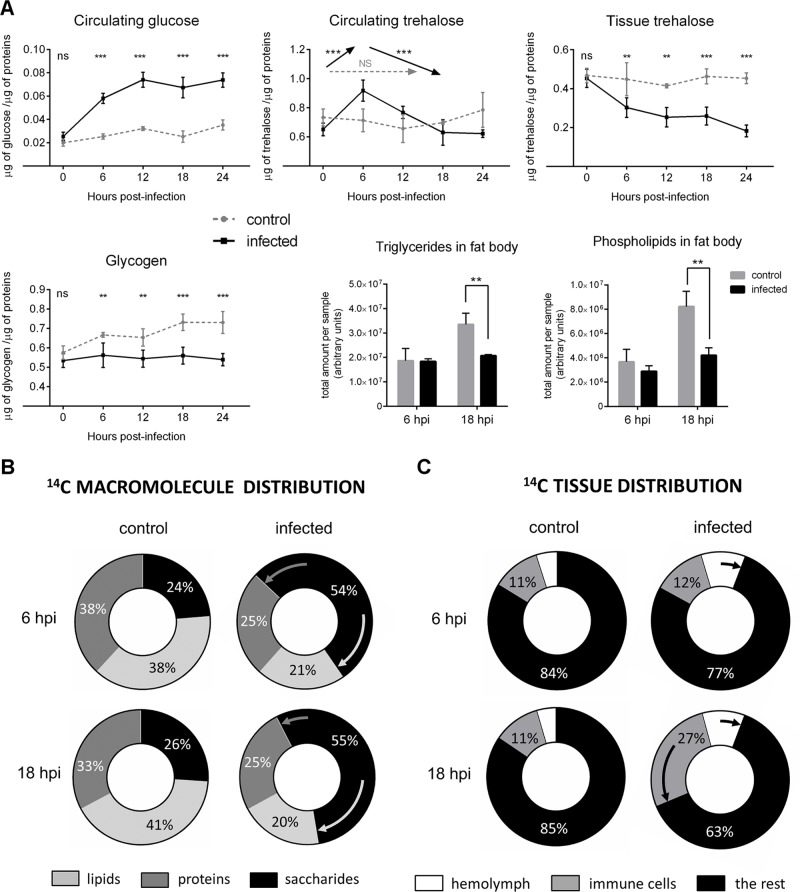
Metabolic changes during immune response in *w* flies. (A) Nutrient contents in the hemolymph, whole larval lysates, and fat body at different time points after infection (uninfected control: grey dashed line and grey column; infected: solid black line and column). Circulating glucose increases, tissue trehalose decreases, glycogen and lipids accumulation ceases upon infection; circulating trehalose first increases and then decreases making a 6 hpi peak. Values are mean ± SEM of four experiments (three for lipids). Asterisks show statistical significance (*p < 0.05; ** *p* < 0.005; *** *p* < 0.0005; ns, not significant) when compared between infected and control samples at indicated time points; arrows (Circulating trehalose, middle) indicate increase, decrease, and no change (NS), respectively, between time points. Significance of differences was tested by two-way ANOVA. (B) Percent incorporation of ^14^C-labeled dietary glucose into lipids, proteins, and saccharides in whole larvae. Incorporation into lipids and proteins decreases upon infection, enlarging saccharide fraction as indicated by arrows. (C) Percent distribution of ^14^C into the hemolymph, immune cells (circulating hemocytes, and lymph gland) and the rest of the larvae (brain, imaginal discs, gut, fat body, and carcass). ^14^C first increases in hemolymph at 6 hpi (from 5% to 10%) and then also in immune cells (from 11% to 27%) at the expense of the rest of the organism upon infection; arrows indicate infection-induced changes. This figure shows data for the *w* genotype; the same values are shown in subsequent figures when compared with other genotypes. See S2 Fig for statistical analysis.

Without infection, circulating glucose was kept below 0.04 μg per μg protein (Fig 2A). Both the glycogen and triacylglycerol (TAG) stores kept increasing, while circulating and tissue trehalose levels remained steady (Fig 2A). Trehalose is a nonreducing disaccharide source of glucose, which is liberated by the action of trehalase [44]. To trace the fate of glucose, we employed dietary radiolabeled D[U-^14^C]-glucose. The glucose-derived ^14^C became evenly distributed in the larvae among saccharides, proteins, and lipids (Fig 2B). About 84% of ^14^C was found in developing tissues (Fig 2C). We divided the organism here in a simplified way into the immune system (represented by cellular immunity, the most important defense against parasitoids, including circulating hemocytes and lymph gland), the circulation (hemolymph), and the rest of the tissues representing mainly development, growth, and energy storage.

**Fig 3:**
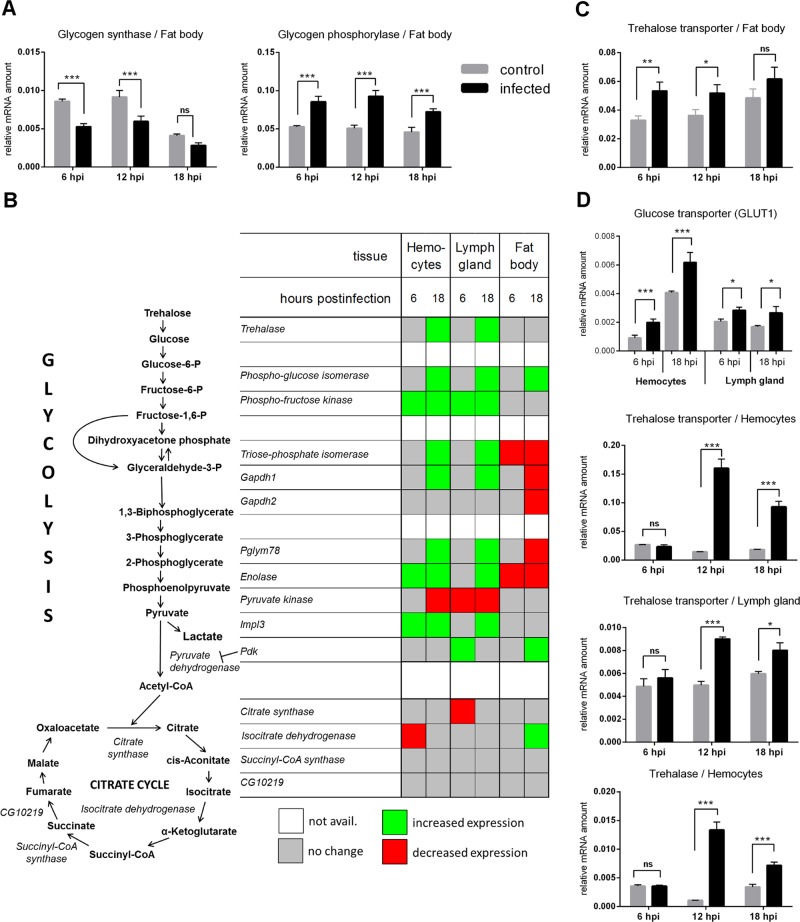
Gene expression during immune response of *w* larvae measured by q-PCR. (A) Reciprocal changes in mRNA expression of glycogen synthase and glycogen phosphorylase enzymes in the fat body. (B) Summary of significant changes in expression of glycolytic and citrate cycle enzymes in the hemocytes, lymph gland, and fat body (see S3–S5 Figs for corresponding graphs). Heat map indicates a tendency of glycolytic genes to increase in immune cells and to decrease in fat body. (C) Expression of trehalose transporter Tret1-1 in the fat body. (D) Expression of GLUT1, TreT1-1, and trehalase in the circulating hemocytes and lymph gland. All graphs except (B) show mean values of expression relative to *Rp49* ± SEM from three independent experiments; grey columns: control larvae, black columns: infected larvae; asterisks indicate significant changes (tested by two-way ANOVA).

In infected larvae, the accumulation of TAG and glycogen reserves ceased (Fig 2A). This was accompanied by down-regulation of glycogen synthase (CG6904; FlyBase ID: FBgn0266064) and up-regulation of glycogen phosphorylase expression (CG7254; FlyBase ID: FBgn0004507)(Fig 3A). The amount of tissue trehalose decreased (Fig 2A), and less dietary glucose was incorporated into lipids and proteins (Fig 2B and S2 Fig). These hallmarks of suppressed energy storage and growth were corroborated by reduced incorporation of ^14^C into developing tissues from 84% in uninfected larvae to 77% at 6 hpi and 63% at 18 hpi (Fig 2C and S2 Fig).

The above effects were associated with hyperglycemia as indicated by elevated hemolymph glucose and ^14^C at the expense of developing tissues (Fig 2A and 2C). Incorporation of ^14^C into lipids and proteins (at the whole organism level) was also suppressed during infection (Fig 2B), which was accompanied by down-regulation of specific glycolytic enzyme genes in the fat body (Fig 3B and S3 Fig). The diversion of metabolism from building energy reserves and from fat body glycolysis was thus in agreement with extra ^14^C in the carbohydrate form and with the increase of circulating glucose and trehalose. Circulating trehalose peaked at 6 hpi (Fig 2A) concomitantly with increased expression of a trehalose transporter in the fat body, the organ where trehalose is produced (Fig 3C).

At the same time, the immune cells changed their behavior during infection in the opposite direction, leading to increased energy consumption. Around one-tenth of ^14^C is normally allocated to immune cells, leaving almost 90% to the rest of the organism, but immune cells demanded up to one-third of nutrients during immune response (Fig 2C). Expression of several glycolytic genes including lactate dehydrogenase *Impl3* (CG10160; FlyBase ID: FBgn0001258) increased both in the circulating hemocytes and in the lymph gland (Figs 3B, S4, and S5). This resembled the glucose-demanding aerobic glycolysis, the Warburg effect, in activated mammalian immune cells. Both the lymph gland and the circulating hemocytes expressed elevated amounts of glucose transporter Glut1 (CG43946; FlyBase ID: FBgn0264574) and trehalose transporter Tret1-1 (CG30035; FlyBase ID: FBgn0050035) mRNAs (Fig 3D). Interestingly, later during infection (12–18 hpi), the circulating hemocytes together with already differentiated lamellocytes strongly increased expression of both Tret1-1 and trehalase (CG9364; FlyBase ID: FBgn0003748) (Fig 3D). This suggests that differentiated immune cells preferentially uptake energy in the form of trehalose, which may be linked to the decline of circulating trehalose after 6 hpi (Fig 2A). These results demonstrate a shift of energy distribution away from storage and growth, first towards circulating glucose and trehalose, and then towards the immune cells (Fig 2).

### e-Ado Signaling via AdoR Is Required for Hyperglycemia and Effective Immune Response

**Fig 4:**
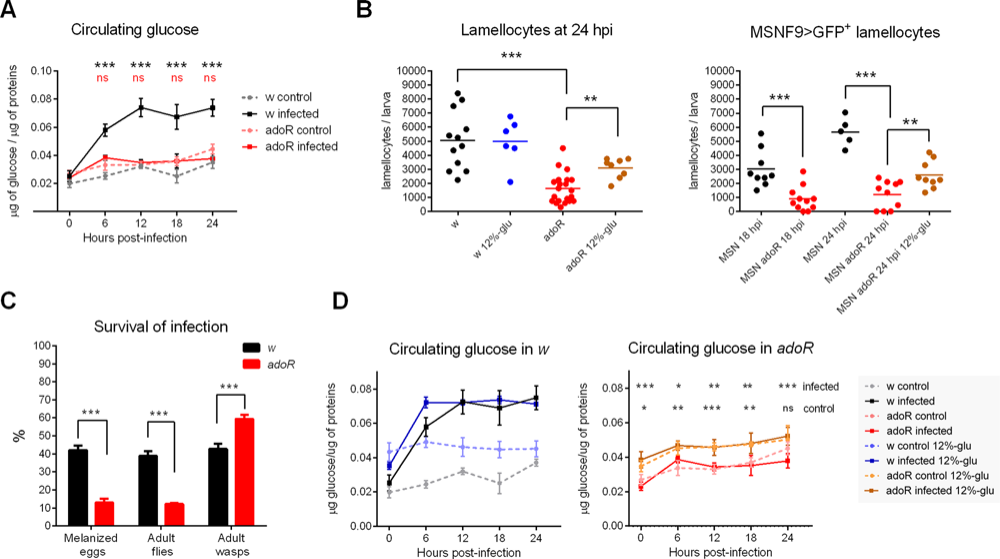
Effects of blocking signaling through *adoR* on immune response. (A) Increase in circulating glucose level during infection is suppressed in the *adoR* mutant. Values are mean ± SEM of four experiments; black asterisks—comparison of *w;* red “ns” (not significant)—comparison of *adoR;* tested by two-way ANOVA. (B) Number of lamellocytes based on cell morphology and a lamellocyte-specific MSNF9>GFP marker. *adoR* larvae contain fewer lamellocytes than *w* or *MSN* controls. High-glucose diet (12%-glu) increases lamellocyte number in *adoR* larvae. Each dot represents lamellocyte count per larva, the lines are mean values; tested by unpaired *t* test. (C) *adoR* mutation significantly reduces the host resistance to parasitoid wasp as assessed from frequency of melanized eggs (*adoR*—13% versus *w*—42%; *n* = 100 *Drosophila* larvae per genotype in five experiments), emerged adult flies (*adoR*—12% versus *w*—38%; *n* = 310 for *adoR*, 316 for *w*, in three experiments). Values are mean ± SEM; tested by unpaired *t* test. (D) High-glucose diet (12%-glu) significantly increases circulating glucose both in uninfected *w* and *adoR* larvae and in infected *adoR* larvae (graph with *w* does not show statistical significance). Values are mean ± SEM of three experiments; tested by two-way ANOVA. In all panels, statistical significance of differences is indicated as *p < 0.05; ** *p* < 0.005; *** *p* < 0.0005; and ns, not significant.

We have previously shown that e-Ado increases circulating glucose via adenosine receptor (AdoR; CG9753; FlyBase ID: FBgn0039747) signaling [38]. Here, we tested if e-Ado was involved in the observed effects of infection on the metabolic shift. While the circulating glucose increased more than 2-fold during infection in *w* larvae, this increase was suppressed in *adoR* (FlyBase ID: FBal0191589) mutant larvae (Fig 4A), indicating that AdoR was indeed necessary for the energy redistribution during infection. Therefore, we compared the number of lamellocytes as a measure of immune response. While *w* larvae produced 5–6 thousand lamellocytes by 24 hpi, the *adoR* mutants contained less than a third of this amount (Fig 4B). Yet the *adoR* mutants were clearly capable of differentiating functional lamellocytes that displayed normal morphology, expressed a lamellocyte-specific MSNF9>GFP marker (FlyBase ID:FBtp0064497), and were capable of encapsulating the wasp egg (Fig 4C and S16 Fig). Therefore, *adoR* larvae were impaired in efficiency or speed of lamellocyte production, and this corresponded with their reduced resistance against the parasitoid invasion relative to *w* larvae. Indeed, the *adoR* mutants were three times less successful at neutralizing the wasp eggs and surviving to adult flies (Fig 4C). Thus, AdoR signaling is crucial for effective immune defense against the parasitoid.

The impaired defense in the *adoR* mutants was not due to affected recognition of the wasp egg, as the number of plasmatocytes attached to the egg surface within the first few hpi was similar in *w* and *adoR* larvae (S7 Fig). Therefore, we tested if shortage of energy could be the problem as suggested by failure to increase circulating sugar levels in *adoR* larvae (Fig 4A). When we fed these larvae a high-glucose diet (12% instead of the regular 5%), the hemolymph glucose significantly increased even without infection in both *w* and *adoR* larvae (Fig 4D). This dietary treatment significantly increased the number of lamellocytes in the infected *adoR* larvae (Fig 4B), suggesting that it was the lack of energy causing inefficient differentiation of lamellocytes in the absence of AdoR.

Interestingly, adding glucose to the diet did not further increase the level of circulating glucose during infection. In fact, the increase induced by infection was greater than that achieved with dietary glucose (Fig 4D), and consistently the number of lamellocytes in infected *w* larvae was the same on both diets (Fig 4B). Since the glucose increase induced by the dietary treatment was not as high as the one induced by the infection, the number of lamellocytes in *adoR* did not reach, even on the high-glucose diet, the levels observed in *w* (Fig 4B). This suggests that the glucose available in circulation is the limiting factor for the lamellocyte differentiation.

### AdoR Signaling Mediates the Metabolic Switch

**Fig 5:**
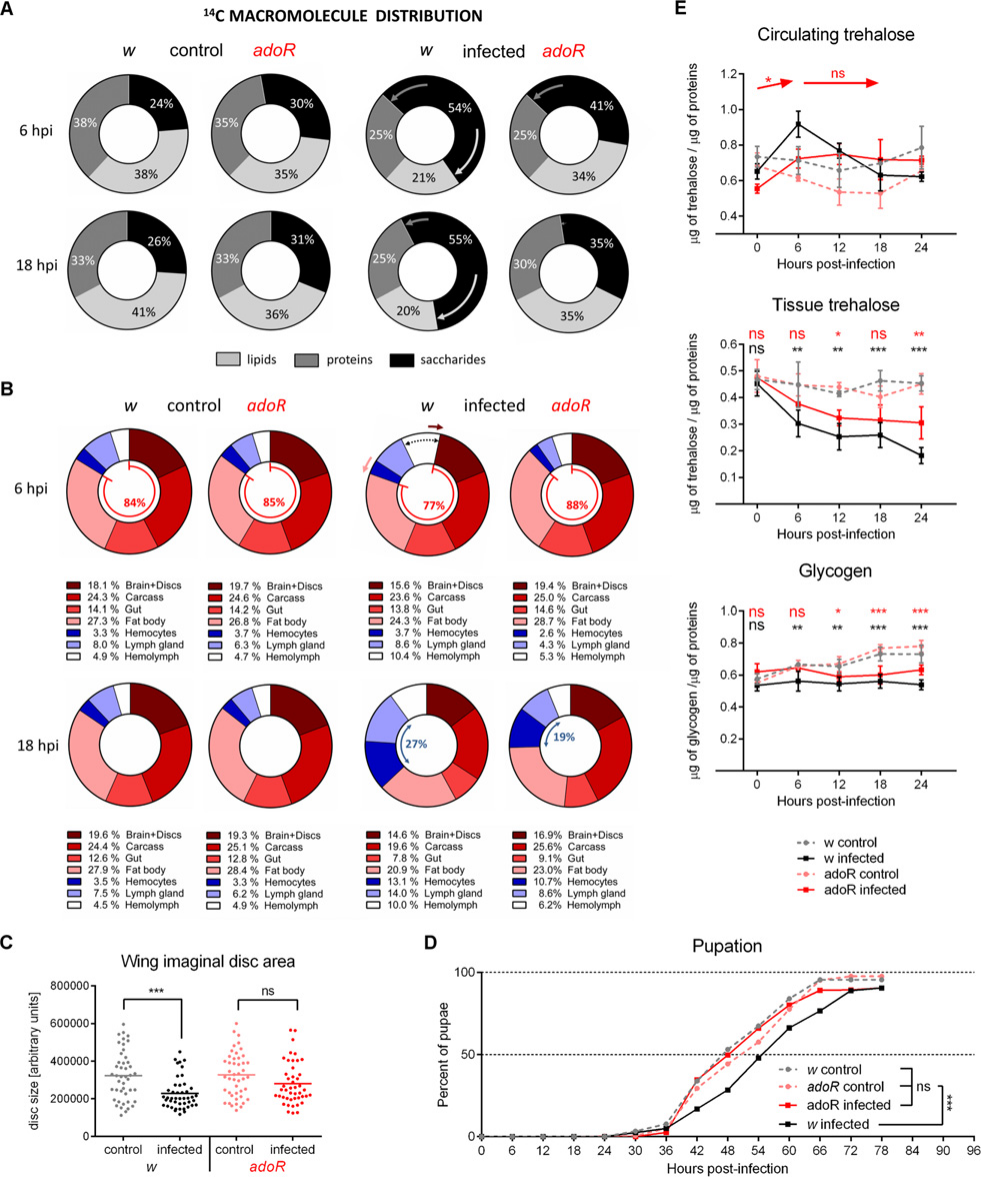
Metabolic changes and developmental effects of AdoR deficiency. (A) Incorporation of ^14^C-glucose into lipids and proteins is reduced upon infection in *w* but not in *adoR* larvae. Arrows indicate infection-induced changes. For statistical analysis, see S2 Fig. (B) Relative distribution of ^14^C in the hemolymph (white), immune cells (circulating hemocytes, dark blue; lymph gland, light blue), and the remaining body parts (brain with imaginal discs—brown; carcass, i.e., all the remnants after dissecting all other presented tissues—red; gut—light red; fat body—pink). Arrows indicate increasing ^14^C in hemolymph (black dashed arrow) of *w* at 6 hpi at the expense of brain+discs (brown arrow) and fat body (pink arrow); these changes are missing in *adoR*. Increase in hemolymph and in immune cells (blue arrow) of *w* at 18 hpi at the expense of all other tissues is smaller in *adoR* (less in immune cells and more in the rest). Legends below graphs show percentages in body parts. For detailed analysis, see S9 Fig and S10 Fig. (C) Growth of the wing imaginal discs is delayed by infection in *w* (unpaired *t* test *p* < 0.0001) but not in *adoR* larvae (*p* = 0.06). Each dot represents measured area of an individual disc at 18 hpi; horizontal lines indicate mean. (D) Pupation is delayed upon infection in *w* larvae (*n* = 316, control and 344, infected) but not in *adoR* larvae (*n* = 310, control and 293, infected). The rates were compared using Log-rank survival analysis; the *p* values are: *w* < 0.0001; *adoR* = 0.74; *w* control versus *adoR* control = 0.053; *w* control versus *adoR* infected = 0.054. (E) Nutrient contents in the hemolymph and whole larval lysates. Values are mean ± SEM of four experiments. Circulating trehalose in *adoR* does not form the 6 hpi peak of *w*; arrows show increase and no change (ns), respectively, when levels of infected *adoR* are compared between time points. Tissue trehalose show smaller differences for *adoR* and glycogen shows similar pattern to *w*. Asterisks show statistical significance when compared between infected and control animals at indicated time points (black for *w*, red for *adoR*). Tested by two-way ANOVA; for statistical analysis, see S2 Fig.

Upon infection, more glucose was retained in the saccharide fraction in the *w* larvae (Fig 2B), indicating that this glucose was available for energy needs of the immune response and less used for storage and growth. Little (at 6 hpi) or no (18 hpi) such retention was observed in *adoR* mutants (Fig 5A and S2 Fig), suggesting that storage and/or growth were not suppressed during infection in the absence of AdoR. This notion was supported by the relative distribution of ^14^C among individual tissues (Fig 5B). The distribution was the same in uninfected *w* and *adoR* animals. The incorporation of ^14^C did not change at 6 hpi in infected *adoR* (as opposed to *w*), and the shift from storage and growth (red part) towards immune cells (blue part) was much smaller in infected *adoR* compared to *w* at 18 hpi (Fig 5B). Importantly, the comparison of relative distribution of ^14^C into tissues was allowed by equal total uptake of ^14^C-glucose from diet in *w* and *adoR* larvae (S8 Fig). Interestingly, the comparison of absolute numbers of ^14^C entering the system also revealed anorexia during infection (lower uptake of ^14^C; S8 Fig), supporting a common observation during immune responses [45]. This anorexia did not seem to depend on AdoR. Besides the lymph gland with slightly lower ^14^C in *adoR* mutants, the tissue distribution of ^14^C was similar in uninfected *w* and *adoR* larvae at both time points (Fig 5B and S10 Fig).

Upon infection, only the brain and imaginal disc complex and fat body of *w* larvae contained significantly less ^14^C while hemolymph contained significantly more ^14^C at 6 hpi (Fig 5B and S9 Fig). While ^14^C incorporation into brain+discs significantly decreased in *w* larvae, it did not change in the *adoR* mutant upon infection (Fig 5B and S9 Fig), demonstrating that the suppression of developmental growth, which occurred during infection, was missing in *adoR*. This is supported by the measurement of the wing imaginal disc growth. While the growth of discs was significantly delayed in *w* control upon infection, the delay did not occur in the *adoR* mutant (Fig 5C). Similarly, the delay in development observed in infected *w* (as measured by pupation rate) did not occur in *adoR*, which pupated as there would be no infection (Fig 5D).

At 18 hpi, all tissues were affected by infection, significantly increasing ^14^C in immune cells and hemolymph and decreasing in the rest (Fig 5B and S9 Fig). In all cases but gut, the changes were significantly smaller in *adoR* than in *w* (S10 Fig), indicating that the AdoR signaling was involved in the overall suppression of nonimmune processes.

The missing suppression of development in *adoR* larvae resulted in shortage of energy available for the immune system as documented first by almost no increase of ^14^C in the hemolymph at 6 hpi and then by much lower ^14^C incorporation into the immune cells at 18 hpi compared to infected *w* larvae (Fig 5B). Weak suppression of nonimmune processes in the absence of AdoR may also be linked to the missing peak of circulating trehalose at 6 hpi (Fig 5E and S2 Fig). Functional AdoR signaling seems to lower glucose transport and to increase trehalose transport in the fat body (suggested by expression levels of the respective transporter genes; S11 Fig), leading to increased trehalose at 6 hpi. The trehalose peak probably serves as a reservoir for fast glucose production, which will be increasingly needed for immune defense. The rapid lamellocyte differentiation is lagging in *adoR* larvae, likely reflecting lower consumption of trehalose relative to *w* larvae (Fig 5E).

### Effect of Ado Transport on Energy Regulation during Immune Response

**Fig 6:**
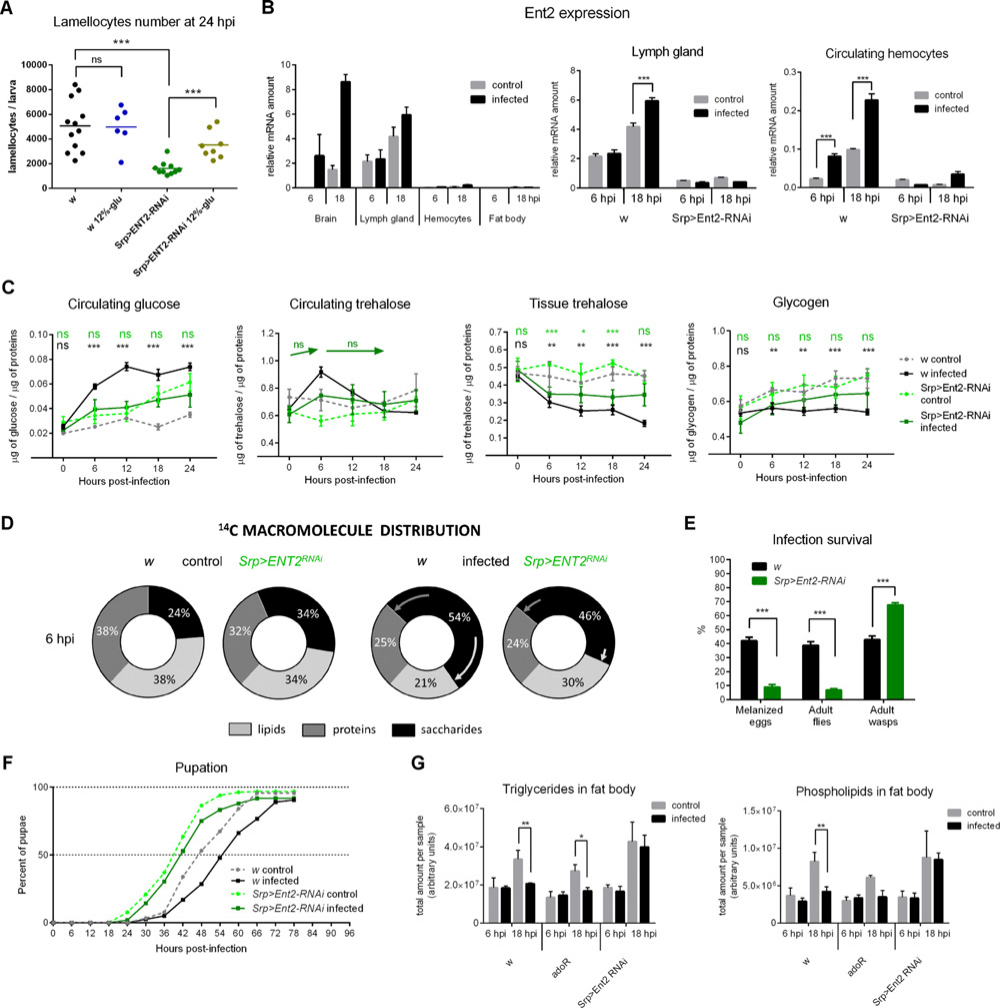
Effects of blocking adenosine transport in immune cells by *ENT2* RNAi. (A) *Srp>ENT2-RNAi* reduces lamellocyte number compared to *w* larvae, and high dietary glucose (12%-glu) significantly increases the lamellocyte number in *Srp>ENT2-RNAi*. Each dot represents lamellocyte count per larva; lines indicate mean. Differences were tested by unpaired *t* test. (B) *ENT2* mRNA expression. Comparison of *ENT2* mRNA expression in various tissues in *w* larvae shows a strong expression in brain and lymph gland, increasing in both upon infection. *Srp>ENT2-RNAi* reduces the *ENT2* expression below 20% both in the lymph gland and hemocytes. Values are mean ± SEM of relative expression (normalized to *Rp49* mRNA) of three experiments; tested by two-way ANOVA. (C) Nutrient contents in the hemolymph and whole larval lysates. Circulating glucose does not increase in *Srp>ENT2-RNAi* upon infection. Circulating trehalose in *Srp>ENT2-RNAi* does not form the 6 hpi peak of *w*; arrows indicate no change (ns) in levels of infected *Srp>ENT2-RNAi* when compared between time points. Tissue trehalose shows similar pattern to *w*. Glycogen does not differ between control and infected *Srp>ENT2-RNAi* indicating an accumulation of stores even upon infection. Asterisks show statistical significance when compared between infected and control animals at indicated time points (black for *w*, green for *Srp>ENT2-RNAi*). Values are mean ± SEM of four experiments; tested by two-way ANOVA. For statistical analysis, see S2 Fig. (D) Incorporation of ^14^C-glucose into lipids and proteins is reduced upon infection in *w* larvae but significantly less so in *Srp>ENT2-RNAi* larvae. Arrows indicate infection-induced changes. For statistical analysis, see S14 Fig. (E) *Srp>ENT2-RNAi* significantly reduces the host resistance to parasitoid wasp as assessed from frequency of melanized eggs (9% versus 42%; *n* = 100 *Drosophila* larvae per genotype in five experiments) and emerged adult flies (7% versus 38%; *n* = 316 for *w*, 343 for *Srp>ENT2-RNAi* in three experiments). Values are mean ± SEM; tested by unpaired *t* test. (F) Uninfected *Srp>ENT2-RNAi* larvae (*n* = 377) pupate 8 h earlier than uninfected *w* larvae, and infection only delays their pupation by 2 h (*n* = 343). Compared using Log-rank survival analysis (*p* < 0.0001 for all comparisons). (G) Total amount of TAG and phospholipids in the fat body of *w*, *adoR*, and *Srp>ENT2-RNAi* larvae. While infection suppresses TAG storage in *w* and *adoR*, TAG grows unaffected by infection in *Srp>ENT2-RNAi*. Data are mean values of mass spectra peak area per sample ± SEM; tested by two-way ANOVA.

The AdoR signaling reallocates energy towards immune defense, suggesting that e-Ado is released upon immune challenge. Therefore, we next wanted to determine the source of e-Ado during wasp invasion. We individually knocked down the Equilibrative nucleoside transporters, *ENT1* (CG11907; FlyBase ID: FBgn0031250) and *ENT2* (CG31911; FlyBase ID: FBgn0263916), which are expressed in *Drosophila* larvae [36,46]. We delivered RNAi to various tissues utilizing the Gal4-UAS system [47], and as a simple readout we used lamellocyte count at 24 hpi (S12 Fig). Among the tested combinations, only *ENT2* knockdown driven by *Srp-Gal4* (FlyBase ID: FBtp0020112) in cells of the hematopoietic lineage achieved a reduction in the number of lamellocytes that resembled the effect of *adoR* deficiency (Figs 4B, 6A, and S12). *Srp-Gal4* was expressed in all hematopoietic cells, including the circulating hemocytes and all cells of the lymph gland that also contained precursors of lamellocytes (S13 Fig). In contrast, knocking down *ENT2* in already differentiated hemocytes (by *Hml-Gal4* and *Upd3*-Gal4 drivers; FlyBase ID: FBtp0040877 and FBtp0020110) did not affect the lamellocyte number (S12 Fig).

*ENT2* mRNA was abundant in the lymph gland and brain but weakly expressed in circulating hemocytes and virtually undetected in the fat body (Fig 6B). During infection, *ENT2* expression increased in all these tissues except the fat body (Fig 6B) and, consistently, *ENT2* RNAi delivered using a fat body-specific *C7-Gal4* driver did not affect the number of lamellocytes (S12 Fig). The increasing expression of *ENT2* during infection in the brain leaves a possibility that the nervous system contributes e-Ado; however, undetectable expression of *Srp-Gal4* in the brain, except for minor signal in some nerve cords (S13 Fig), makes the observed effects of ENT2 removal attributable to the immune cells.

The results above suggest that Ado transport from immune cells, including the differentiating ones, is important for efficient lamellocyte differentiation. As in the case of *adoR* mutation (Fig 4B), the loss of lamellocytes was rescued by increasing dietary glucose in the *Srp>ENT2-RNAi* larvae (Fig 6A). Similarly to *adoR* mutation, *ENT2* knockdown in immune cells also cancelled changes in nutrient distribution that normally take place in infected *w* larvae; there was no peak of circulating trehalose at 6 hpi and no increase in circulating glucose (Fig 6C and S2 Fig). The partition of ^14^C into saccharides, proteins, and lipids also resembled the pattern seen in *adoR* mutant larvae (compare Fig 6D with Fig 5A and S2 Fig with S14 Fig).

Together, the above data indicate that deficiency in e-Ado release and in its receptor, AdoR, consistently lead to the same failure of energy reallocation during immune challenge. Indeed, like loss of AdoR, knocking down *ENT2* also reduced the host resistance against wasp invasion (Fig 6E), while the normal developmental delay observed in *w* controls upon infection did not occur in *Srp>ENT2-RNAi* larvae (Fig 6F). Interestingly, pupation occurred earlier in *Srp>ENT2-RNAi* compared to *w* or *adoR* animals even without infection (Fig 6F); the size of pupae was unaffected implying faster growth instead of precocious pupation of *Srp>ENT2-RNAi*.

While glycogen storage was suppressed similarly upon infection in *adoR* mutant and *w* larvae (Fig 5E), there was no significant difference in glycogen content between infected and uninfected *Srp>ENT2-RNAi* larvae (Fig 6C). Even more apparent was the effect on lipid storage where the accumulation of TAG in the fat body was suppressed both in *w* and *adoR* but not at all in *Srp>ENT2-RNAi* larvae (Fig 6G). Blocking Ado transport thus led to continued nutrient storage even upon immune challenge, suggesting that energy storage during infection might be regulated by e-Ado independently of AdoR.

## Discussion

**Fig 7:**
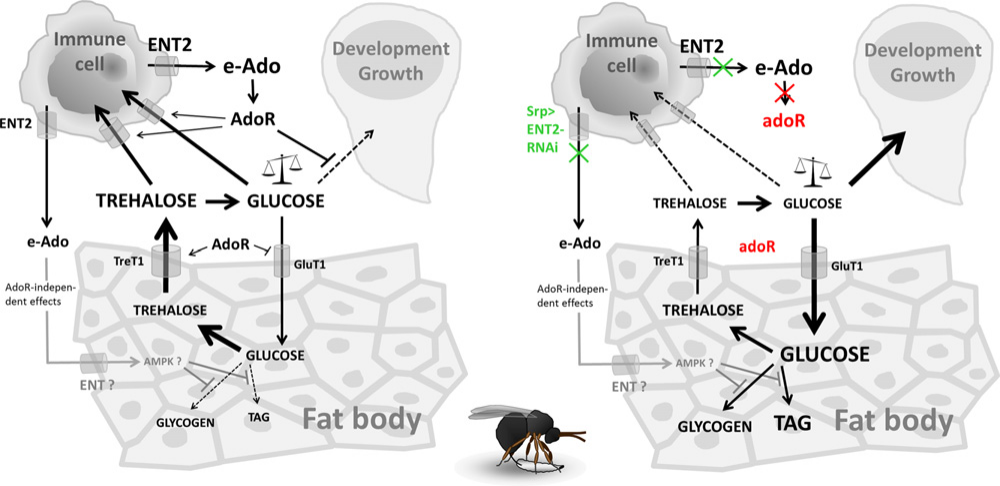
Model of metabolic shifts mediated by e-Ado during immune response. Left—wild-type situation upon infection. Right—situation without e-Ado upon infection; blocking AdoR signaling by *adoR* mutation is marked in red, blocking Ado transport from immune cells by *Srp>ENT2-RNAi* is marked in green. See text for details.

An overall metabolic suppression is a common host response to infection [18,12,6]. A likely purpose for the suppression is to conserve energy for the immune response that is energetically costly [2,12]. The defense of the *Drosophila* larva against the parasitoid wasp requires a rapid production of specialized immune cells (lamellocytes) that encapsulate the parasitoid egg. This has provided us with a unique in vivo model to study the metabolic changes and their regulation during immune response. We show here that the production of lamellocytes is an energetically demanding process, and that a systemic metabolic switch is required for their effective differentiation. This switch includes (1) suppression of energy storage and developmental growth, (2) retaining more energy in circulation, and (3) increased consumption of energy by the immune system (Fig 7).

Suppression of energy storage (glycogen and lipids) and suppression of growth, as documented by slower growth of imaginal discs, lead to a developmental delay. We show here that e-Ado is a signal mediating this metabolic switch. Blocking this signal then demonstrates that the metabolic switch is crucial for an effective immune response. Without this signal, development and growth proceed at a normal speed, thus reducing energy available to the immune cells. Insufficiency of immune cells due to the shortage of energy then leads to a drastically reduced resistance against the parasitoid. Experimental interference with e-Ado or its receptor, AdoR, thus demonstrates the importance of tradeoff between development and immune response, and identifies e-Ado as a signal responsible for the switch.

Blocking Ado transport from immune cells by knocking down the equilibrative nucleoside transporter ENT2 identified the differentiating immune cells as an important source of the signal for the metabolic switch. This suggests that the immune cells could autonomously regulate energy influx based on their acute needs. Ado is a fine sensor of the cellular energy state, as it becomes produced when the ATP:AMP ratio decreases [23]. This scenario is appealing mainly because immune cells dramatically change their metabolism upon activation, leading to increased aerobic glycolysis akin to the Warburg effect [3,4]. Our expression analysis of glycolytic genes, glucose and trehalose transporters, and ^14^C uptake by immune cells suggested a similar behavior for the differentiating immune cells upon wasp attack. The ability to rapidly react to a metabolic stress could be why ENT2 is strongly expressed in the lymph gland and the brain, both privileged organs from the energy point of view.

AdoR signaling is important for the suppression of developmental growth. Normally, infection leads to lower consumption of energy by the brain and imaginal discs (later also by other tissues), but the consumption continues in *adoR*-deficient larvae as if they were uninfected. At the same time, AdoR signaling seems to lower glucose transport and to increase trehalose transport in the fat body as inferred from expression levels of the respective transporter genes. The fat body is the site where trehalose is produced from glucose [44]; trehalose is then released back to the hemolymph, and more so during infection. The *adoR* mutation causes a misbalance of glucose and trehalose transport in the fat body, causing more nutrients to be retained there. The effect of AdoR signaling on the fat body combined with the suppression of developmental growth leads to hyperglycemia that in turn ensures enough energy to supply the immune cells. If the growth suppression fails to occur, as in the *adoR* mutant, the immune cells are unable to compete with developing tissues that consume the majority of energy. By analogy to the selfish brain theory [48], “selfish” immune cells may usurp energy to themselves by way of AdoR-mediated silencing of nonimmune processes. Our work thus brings experimental evidence and explains the molecular mechanism for recently published theoretical concept of selfish immune system [49].

Interestingly, the AdoR signaling does not mediate the suppression of energy storage (glycogen and TAG) during infection. However, increasing glycogen and TAG stores in infected *Srp>ENT2-RNAi* larvae with blocked Ado transport from immune cells indicates that the storage suppression is also under e-Ado control but through an AdoR-independent mechanism. Such a mechanism, which needs to be further studied, may involve e-Ado uptake, conversion to AMP by adenosine kinase, and activation of AMPK [25]. The *Srp>ENT2-RNAi* larvae proceeded faster through development not only during infection but even without infection when compared to control larvae. This suggests that the regulation of energy storage by e-Ado may play a role even during normal development.

e-Ado signaling was previously associated with regulation of hemocyte differentiation, and blocking the AdoR signaling was suggested to lower the differentiation in the lymph gland under noninfectious conditions [50]. The hallmark of lamellocyte differentiation upon parasitoid wasp infection is the turning off the Jak-Stat signaling in the medullary zone of the lymph gland containing the prohemocytes [51]. Expression of cytokine Upd3 (CG33542; FlyBase ID: FBgn0053542) is down-regulated, and the ratio of Jak-Stat receptor Domeless (CG14226; FlyBase ID: FBgn0043903) and its negative coreceptor Latran (CG14225; FlyBase ID: FBgn0031055) is switched upon wasp infection leading to turning off the Jak-Stat and to induction of lamellocyte differentiation [52]. The expression patterns of *Upd3*, *Domeless* and *Latran* mRNAs normally and during infection are unaffected both in *adoR* and *Srp>ENT2-RNAi* (S15 Fig), indicating that the induction of lamellocyte differentiation is functional in these lines. In addition, the lymph glands develop normally in both *adoR* and *Srp>ENT2-RNAi* ([50] and S17 Fig). Our results demonstrate that the *adoR* and *Srp>ENT2-RNAi* larvae are capable of lamellocyte differentiation; they are just less effective, and the reason is most likely the lack of energy as indicated by the rescue of this phenotype with extra dietary glucose.

An important part of the global energy switch observed upon parasitoid invasion is the AdoR-mediated suppression of developmental growth. Although AdoR is relatively strongly expressed in imaginal discs [34], we do not know if it is the tissue-autonomous signaling of AdoR, or whether AdoR acts systemically on metabolism as AdoR is also strongly expressed in the larval endocrine glands and brain; both scenarios may apply simultaneously. It is known that the activation of adenosine receptor leads to metabolic suppression—at the individual cell level, the activation can inhibit growth of tumor cells [26], but it can also cause a systemic suppression during anoxia [28,29] or torpor [27]. Our work demonstrates that the AdoR-mediated suppression plays an important role also during immune response. It will be important to identify the target cells and signaling cascades mediating the observed suppression in future studies.

We show here that the metabolic switch is mediated by e-Ado and that the switch is crucial for an effective immune response. It is of interest to see if this e-Ado role is common to other organisms including humans. e-Ado plays the same role in energy regulation in flies and mammalian systems [30,38]. For example, sepsis is associated with hyperglycemia and insulin resistance as well as with increased e-Ado [31,53], suggesting that e-Ado could indeed mediate the systemic metabolic switch in higher organisms. However, analyzing this role of e-Ado in mammals will be complicated by the existence of multiple adenosine receptors with partly contradicting functions [54,55] and by diverse roles of e-Ado in immunomodulation [24,56,57].

In conclusion, our study demonstrates that extracellular adenosine, released from immune cells, mediates a systemic metabolic switch leading to suppression of energy storage and developmental growth, thus leaving more energy to the immune cells. This switch is crucial for the effective immune response and blocking adenosine signaling drastically reduces host resistance to the pathogen. This may resemble a selfish brain theory in a way that the immune system, like the brain, is a privileged part of the organism, capable of suppressing energy consumption by other tissues in its own interest. Such a selfish immune system [49] would use e-Ado as a signal to appropriate extra energy resources during immune challenge.

## Materials and Methods

### Fly Stocks, Culture, and Infection

All strains were backcrossed at least ten times to *w* ^*1118*^ genetic background; *w* ^*1118*^ was used as a control in all experiments. *adoR* mutant was homozygous for *adoR* ^*1*^ mutation (FBal0191589). RNAi lines originated from VDRC: *UAS-Ent1-RNAi* (ID 109885) and *UAS-Ent2-RNAi* (ID 100464). *SrpD-Gal4*, *Upd3-Gal4*, and *MSNF9-GFP* were obtained from Michele Crozatier, *HmlΔ-Gal4* from Bruno Lemaitre and *C7-Gal4* from Marek Jindra. Flies were grown on cornmeal medium (8% cornmeal, 5% glucose, 4% yeast, 1% agar) at 25°C. For dietary treatment, larvae were transferred upon infection to cornmeal diet with 12% instead of 5% glucose. Early 3rd instar larvae were infected by parasitoid wasp *L*. *boulardi*. Weak infection (1–2 eggs per larva) was used for resistance and pupation analysis; strong infection (4–7 eggs per larva) was used in all other cases.

### Resistance and Pupation

To determine pupation rate and resistance to parasitoids, infected and control larvae were placed into fresh vials (1 experiment = 30 larvae per vial, 3 vials per genotype; 4 independent experiments). Pupation rate was determined by counting newly appeared pupae every 6 h and incremental percentage of number of pupae per total number of infected and control larvae at a particular time point postinfection was plotted; Log-rank survival analysis was used for comparison. For resistance, we first dissected 20 larvae per experiment from each genotype to count fully melanized wasp eggs (winning host) or surviving wasp larvae (winning parasitoid). Second, we counted all emerged adult flies as surviving the infection and flies without any egg (i.e., uninfected individuals) were excluded from the total number in the experiment. Adult wasps emerged from the vial were counted as adult parasitoid winners.

### Gene Expression

Expression was analyzed by quantitative real-time PCR. Samples were collected from three independent infection experiments with three technical replicates for each experiment. Expression was normalized to Ribosomal protein *Rp49*.

### ^14^C-glucose Distribution

Larvae were fed either 73 h AEL or 91 h AEL for 20 min a diet containing D[U-^14^C]-glucose (10.6 Gbq/mmol; Amersham Biosciences) in yeast. Samples were collected 5 h later. Each sample contained tissues from 30 larvae—all hemolymph was collected by ripping larvae in PBS, centrifuging them, and dividing them into pelleted hemocytes and hemolymph fractions; brains with attached discs and wing discs, whole guts, whole fat bodies, and lymph glands were separated by dissection, and the rest were used as carcass. Macromolecular fractions were separated from tissue homogenates according to [58] for saccharides and lipids and by TCA treatment for proteins. Part of the homogenate was used for measurement of total absorbed amount of ^14^C molecules. Number of ^14^C disintegrations per minute was detected by liquid scintillator.

### Metabolites Measurement

Glucose, trehalose, and glycogen were measured as described [59], using GAGO-20 kit (Sigma). Lipids extracted with chlorophorm:methanol were quantified by HPLC and mass spectrometry.

### Imaginal Disc Size Measurement

Wing discs were dissected from larvae at 90 h AEL (18 hpi), and their size was determined from micrographs by FIJI software.

### Data Analysis

Data were analyzed by GraphPad Prism 6 (GraphPad Software, Inc.).

Extended Materials and Methods are available in S1 Text.

## Supporting Information

S1 Data Compressed ZIP file containing 17 dataset files with original data in GraphPad PRISM 6 format. Fig 1 —data.pzfx: Immune response to parasitoid wasp intrusion. Fig 2 —data.pzfx: Metabolic changes during immune response in *w* flies. Fig 3 —data.pzfx: Gene expression during immune response of *w* larvae measured by q-PCR. Fig 4 —data.pzfx: Effects of blocking signaling through adenosine receptor (*adoR*) on immune response. Fig 5 —data.pzfx: Metabolic changes and developmental effects of AdoR deficiency. Fig 6 —data.pzfx: Effects of blocking adenosine transport in immune cells by *ENT2* RNAi. S2 Fig—data.pzfx: Statistical analysis of metabolite changes. S3 Fig—data.pzfx: Gene expression analysis of glycolytic and citrate cycle genes in fat body by q-PCR. S4 Fig—data.pzfx: Gene expression analysis of glycolytic and citrate cycle genes in circulating hemocytes by q-PCR. S5 Fig—data.pzfx: Gene expression analysis of glycolytic and citrate cycle genes in lymph gland by q-PCR. S7 Fig—data.pzfx: Recognition of parasitoid wasp egg by plasmatocytes is not affected by *adoR*. S8 Fig—data.pzfx: Total absorption of 14C from food. S9–S10 Fig—data.pzfx: Comparison of relative ^14^C-tissue distribution between infected and uninfected larvae and between *w* and *adoR*. S11 Fig—data.pzfx: q-PCR expression analysis of genes involved in transport and metabolism of glucose and trehalose in *w* and *adoR*. S12 Fig—data.pzfx: Number of lamellocytes in flies with knocked-down equilibrative nucleoside transporters ENT1 and ENT2 in different tissues. S14 Fig—data.pzfx: Relative ^14^C incorporation into macromolecules in *w* and Srp>ENT2-RNAi at 6 hpi. S15 Fig—data.pzfx: Expression analysis of genes involved in regulation of lamellocytes differentiation by q-PCR.
(ZIP)

S1 Fig Timescale of experimental procedures. Top timescale—sampling and treatment description for experiments with ^14^C-labeled glucose. Middle timescale—sample collection and treatment for experiments characterizing reaction to infection. Bottom timescale—sample collection for experiments characterizing metabolites.
(TIF)

S2 Fig Statistical analysis of metabolite changes. A) Statistical analysis of infection-induced changes in circulating trehalose between infection and control in particular time points (upper table) and between different time points either in control or infection (lower table); tested by two-way ANOVA, B) comparison of incorporation of ^14^C into macromolecules (saccharides, lipids, and proteins), and C) into three distinguished processes (development and growth, cellular immunity, and circulation) in *w* and *adoR*; tested by two-way ANOVA. Uninfected individuals marked as CON (grey columns), infected individuals marked as INF (black columns). Graphs show mean values ± SEM of three independent experiments. Asterisks show statistical significance (*<0.05; **<0.005; ***<0.0005).
(TIF)

S3 Fig Gene expression analysis of glycolytic and citrate cycle genes in fat body by q-PCR. Infection-induced difference in gene expression was analyzed in *w* and *adoR*, 6 and 18 hpi. Uninfected individuals are represented by grey columns (CON), infected individuals by black columns (INF). Graphs show mean values relative to Rp49 ± SEM of three independent experiments. Asterisks show statistical significance (*<0.05; **<0.005; ***<0.0005); tested by one-way ANOVA. Gene symbols and the corresponding genes can be found in S1 Table.
(TIF)

S4 Fig Gene expression analysis of glycolytic and citrate cycle genes in circulating hemocytes by q-PCR. Infection-induced difference in gene expression was analyzed in *w* and *adoR*, 6 and 18 hpi. Uninfected individuals are represented by grey columns (CON), infected individuals by black columns (INF). Graphs show mean values relative to Rp49 ± SEM of three independent experiments. Asterisks show statistical significance (*<0.05; **<0.005; ***<0.0005); tested by one-way ANOVA. Gene symbols and the corresponding genes can be found in S1 Table.
(TIF)

S5 Fig Gene expression analysis of glycolytic and citrate cycle genes in lymph gland by q-PCR. Infection-induced difference in gene expression was analyzed in *w* and *adoR*, 6 and 18 hpi. Uninfected individuals are represented by grey columns (CON), infected individuals by black columns (INF). Graphs show mean values relative to Rp49 ± SEM of three independent experiments. Asterisks show statistical significance (*<0.05; **<0.005; ***<0.0005); tested by one-way ANOVA. Gene symbols and the corresponding genes can be found in S1 Table.
(TIF)

S6 Fig Comparison of glycolytic and citrate cycle genes expressions in *w* and *adoR* summarized by heat map. Comparison of infection-induced differences in gene expression between w and *adoR* in three different tissues (hemocytes, lymph gland, and fat body) and two different time points postinfection (6 and 18 hpi). Green squares—increased expression, red squares—decreased expression, grey squares—no significant difference, white squares—not analyzed. Level of significance *p* < 0.05; one-way ANOVA.
(TIF)

S7 Fig Recognition of parasitoid wasp egg by plasmatocytes is not affected by *adoR*. (A) Percentage of eggs with certain number of plasmatocytes (none, 1–10, 10–20, or >20) attached to their surface within the first 2 hpi and 4–6 hpi in *w* and *adoR* mutant larvae. (B) Examples of attached Hml>GFP-labeled hemocytes (green fluorescence) to parasitoid wasp egg within the first 2 hpi and 4–6 hpi in *w* and *adoR* mutant larvae.
(TIF)

S8 Fig Total absorption of ^14^C from food. Larvae were fed ^14^C-glucose (in blue dye-labeled diet) for 20 min and then transferred to normal diet for 5 h in which they absorbed ^14^C-glucose and cleared their guts. They were then homogenized to analyze how much ^14^C-glucose they absorbed. There is no difference in absorption between control *w* and control *adoR* or infected *w* and infected *adoR* both at 6 and 18 hpi (labeled NS for not significant). Interestingly, infected larvae (both *w* and *adoR*) absorbed less ^14^C than control larvae indicating anorexia upon infection. Graph shows uninfected *w* (grey columns), infected *w* (black columns), uninfected *adoR* (pink columns), and infected *adoR* (red columns) mean values of disintegration of ^14^C per minute (dpm) per sample ± SEM of three independent experiments, tested by one-way ANOVA. Asterisks show statistical significance (*<0.05; **<0.005; ***<0.0005).
(TIF)

S9 Fig Comparison of relative ^14^C-tissue distribution between infected and uninfected larvae. This figure serves as an alternative for Fig 5B to visualize statistical significance. Compared values: uninfected *w* (grey columns) with infected *w* (black columns) and uninfected *adoR* (pink columns) with infected *adoR* (red columns). Graph shows mean values ± SEM of three independent experiments. Tested by one-way ANOVA with Arc-Sin transformation. Asterisks show statistical significance (*<0.05; **<0.005; ***<0.0005).
(TIF)

S10 Fig Comparison of relative ^14^C-tissue distribution between *w* and *adoR*. This figure serves as an alternative for Fig 5B to visualize statistical significance. Compared values: uninfected *w* (grey columns) with uninfected *adoR* (pink columns) and infected *w* (black columns) with infected *adoR* (red columns). Graph shows mean values ± SEM of three independent experiments. Tested by one-way ANOVA with Arc-Sin transformation. Asterisks show statistical significance (*<0.05; **<0.005; ***<0.0005).
(TIF)

S11 Fig q-PCR expression analysis of genes involved in transport and metabolism of glucose and trehalose in *w* and *adoR*. Graphs display infection-induced differences in expression level of Trehalose transporter TreT1-1, Glucose transporter 1 (Glut1), Glycogen synthase (Gsyn), and Glycogen phosphorylase (Gps) in fat body, lymph gland, and circulating hemocytes at 6 and 18 hpi. Uninfected individuals marked as CON (grey columns), infected individuals marked as INF (black columns). Graph shows mean values ±SEM of three independent experiments. Asterisks show statistical significance (*<0.05; **<0.005; ***<0.0005; ns for nonsignificant difference); tested by one-way ANOVA.
(TIF)

S12 Fig Number of lamellocytes in flies with knocked-down equilibrative nucleoside transporters ENT1 and ENT2 in different tissues. RNAi was induced by driving *UAS-Ent1-RNAi* (VDRC ID 109885) and *UAS-Ent2-RNAi* (VDRC ID 100464) by various Gal4 drivers: *Srp-Gal4* expressed in all cells of hematopoietic lineage and fat body, *Upd3-Gal4* and *Hml-Gal4* in differentiated hemocytes and *C7-Gal4* in fat body. Lamellocytes were counted based on morphology using DIC at 24 hpi. Only combination of *Srp>Ent2-RNAi* significantly decreased lamellocytes. Results were tested by one-way ANOVA, each point in graph represents number of lamellocytes in one individual larva. Asterisks show statistical significance (*<0.05; **<0.005; ***<0.0005; NS for nonsignificant difference).
(TIF)

S13 Fig Expression of SrpD-Gal4 driver in 72-h-old larvae. *Srp-Gal4* driver expression was visualized by crossing to *UAS-GFP*. Strong expression was detected in all cells of hematopoietic lineage as demonstrated by the GFP fluorescence in circulating hemocytes and all cells in the lymph gland. The expression was also detected in fat body but it was undetectable in the brain besides weak expression in nerve cords. Left panels show DIC image corresponding to GFP fluorescence images on right.
(TIF)

S14 Fig Relative ^14^C incorporation into macromolecules in *w* and Srp>ENT2-RNAi at 6 hpi. Comparison of incorporation of ^14^C into macromolecules (saccharides, lipids, and proteins) tested by two-way ANOVA. Uninfected individuals are marked as control (grey columns), infected individuals are marked as infected (black columns). Graphs show mean values ± SEM of three independent experiments. Asterisks show statistical significance (*<0.05; **<0.005; ***<0.0005; ns for not significant).
(TIF)

S15 Fig Expression analysis of genes involved in regulation of lamellocytes differentiation by q-PCR. RNA was isolated from dissected lymph glands at 6 hpi of control (grey) and infected (black) larvae. Graphs show the reciprocal changes in expression of Jak-Stat receptor *Domeless* and its negative coreceptor *Latran* and turning off *Unpaired3* (*Upd3*) upon infection. This hallmark of induction of lamellocyte differentiation does not differ in *adoR* or *Srp>ENT2-RNAi* compared to *w*. Graph shows mean values ± SEM of three independent experiments. Differences were tested by one-way ANOVA; results shown below the graphs—ns = not significant; *<0.05; **<0.005; ***<0.0005; ****<0.00005).
(TIF)

S16 Fig Expression of lamellocyte-specific MSNF9>GFP marker. (A) Expression of MSNF9>GFP (green) together with plasmatocytes-specific P1 marker (red) in the lymph gland upon infection at 12 and 18 hpi. Both *w* and *adoR* express the lamellocyte marker indicating that *adoR* is able to differentiate lamellocytes but there is usually less MSNF9>GFP signal in *adoR* at 12 hpi. While *w* sometimes already releases lamellocytes into circulation at 18 hpi (as demonstrated by disintegrated lymph gland in DIC picture), *adoR* has lymph gland still compact at 18 hpi, but with increasing number of MSNF9>GFP positive cells further demonstrating ability of *adoR* to differentiate lamellocytes but with lower speed. Top—DIC, bottom—fluorescence confocal image. (B) MSNF9>GFP positive lamellocytes in circulation at 27 hpi are present in both *w* and *adoR* and their morphology is indistinguishable. Fluorescence and DIC-combined micrographs.
(TIF)

S17 Fig Morphology of the lymph gland in 72-h-old larvae. Morphology and zonation of the lymph gland in 72-h-old larvae (corresponding to time of infection) was checked by expression of medullary zone-specific *Dome>GFP* marker (prohemocyte containing zone; green) and differentiated plasmatocyte-specific P1 marker for cortical zone (red) in *w* and *adoR*. Only P1 marker was used in *Srp>ENT2-RNAi* and DAPI (nuclear staining) for overall morphology to define medullary zone by the absence of P1. In all three genotypes, the zonation and morphology was comparable for multiple samples indicating that there is no gross effect of the used genetic manipulations on the lymph gland development prior to infection.
(TIF)

S1 Table Genes, gene symbols, and primers used for q-PCR analysis. (XLSX)

S1 Text Extended materials and methods. (DOCX)

## Abbreviations

ADGF-A: adenosine deaminase-related growth factor A
AdoR: adenosine receptor
AMPK: AMP-activated protein kinase
e-Ado: extracellular adenosine
hpi: hours postinfection
SEM: standard error of measurement
TAG: triacylglycerol
