# Supplementary figures and images for: Extracellular Adenosine Mediates a Systemic Metabolic Switch during Immune Response
Source: PLoS Biol. 2015 Apr 27;13(4):e1002135. doi: 10.1371/journal.pbio.1002135 (PMC4411001; doi:10.1371/journal.pbio.1002135)

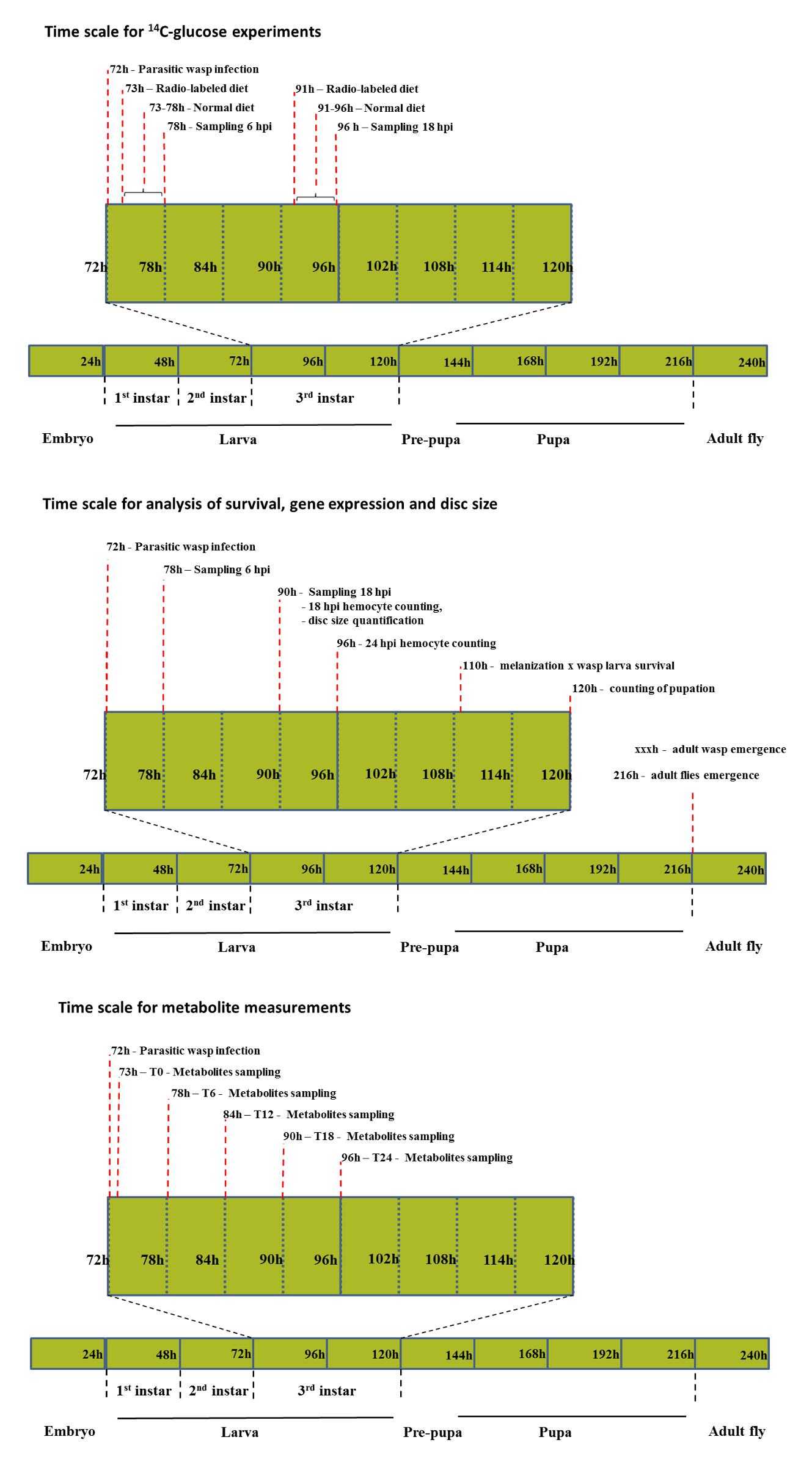

Supplement: S1 Fig — Top timescale—sampling and treatment description for experiments with 14C-labeled glucose. Middle timescale—sample collection and treatment for experiments characterizing reaction to infection. Bottom timescale—sample collection for experiments characterizing metabolites. (TIF) [file pbio.1002135.s002.tif]

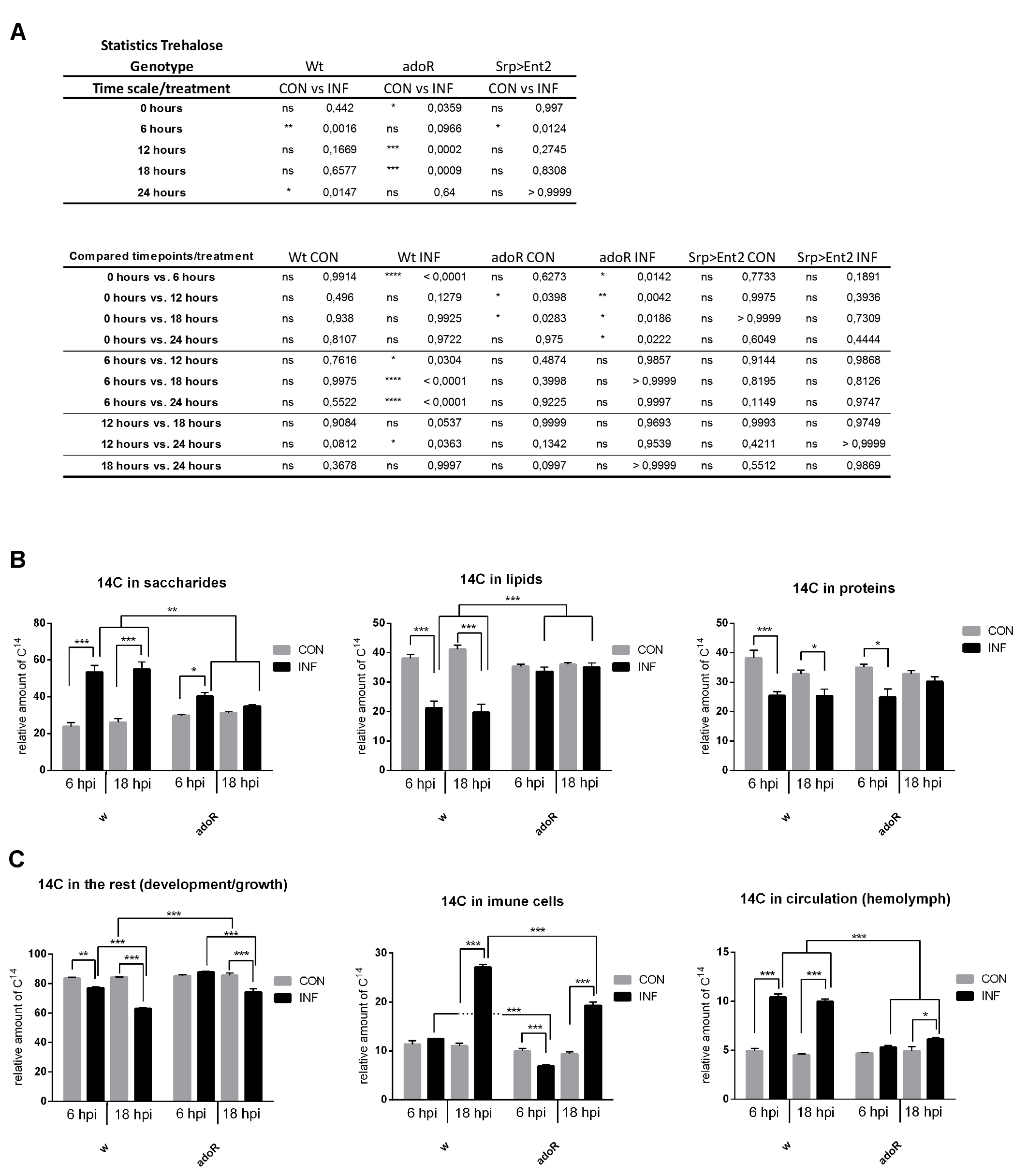

Supplement: S2 Fig — A) Statistical analysis of infection-induced changes in circulating trehalose between infection and control in particular time points (upper table) and between different time points either in control or infection (lower table); tested by two-way ANOVA, B) comparison of incorporation of 14C into macromolecules (saccharides, lipids, and proteins), and C) into three distinguished processes (development and growth, cellular immunity, and circulation) in w and adoR; tested by two-way ANOVA. Uninfected individuals marked as CON (grey columns), infected individuals marked as INF (black columns). Graphs show mean values ± SEM of three independent experiments. Asterisks show statistical significance (*<0.05; **<0.005; ***<0.0005). (TIF) [file pbio.1002135.s003.tif]

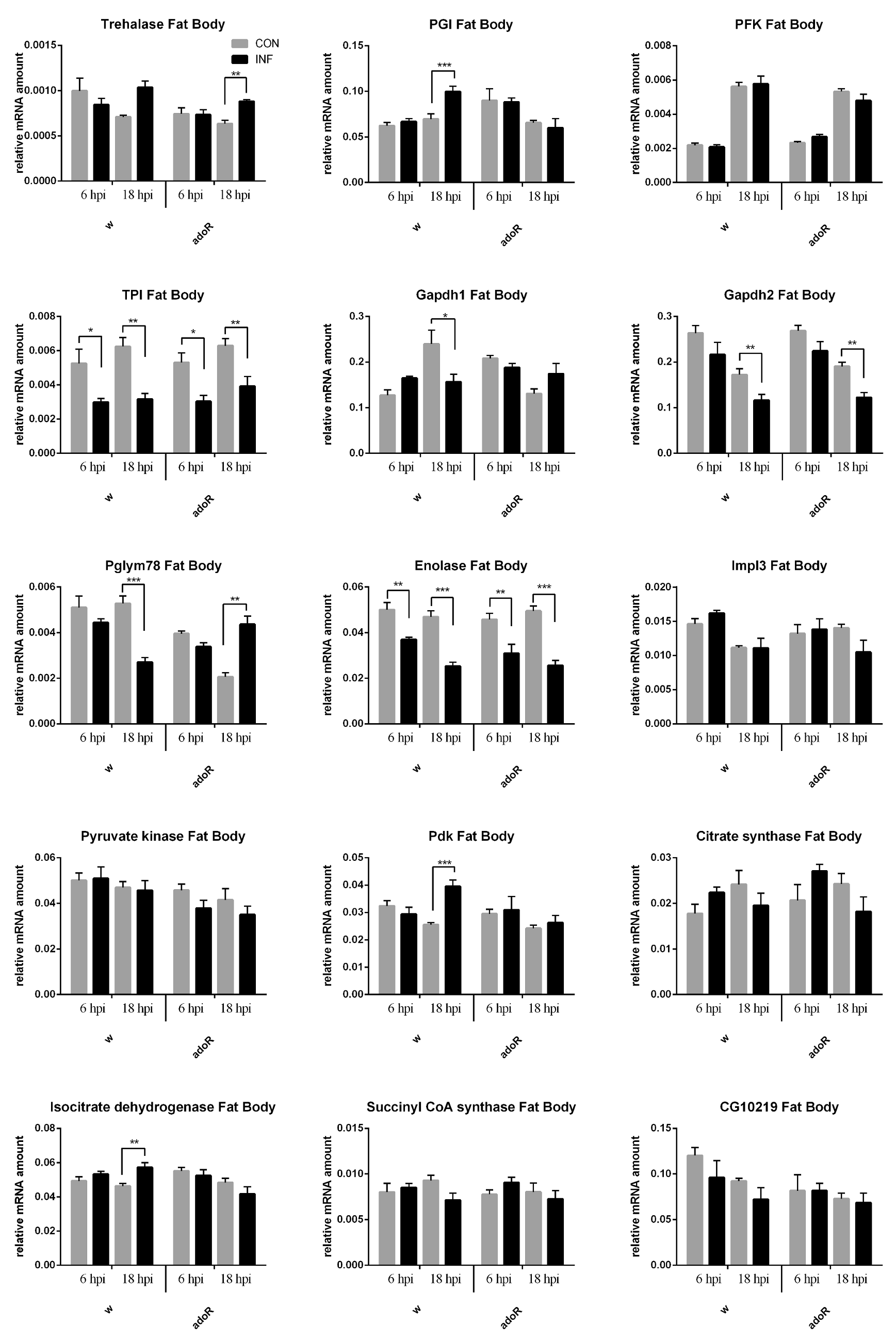

Supplement: S3 Fig — Infection-induced difference in gene expression was analyzed in w and adoR, 6 and 18 hpi. Uninfected individuals are represented by grey columns (CON), infected individuals by black columns (INF). Graphs show mean values relative to Rp49 ± SEM of three independent experiments. Asterisks show statistical significance (*<0.05; **<0.005; ***<0.0005); tested by one-way ANOVA. Gene symbols and the corresponding genes can be found in S1 Table. (TIF) [file pbio.1002135.s004.tif]

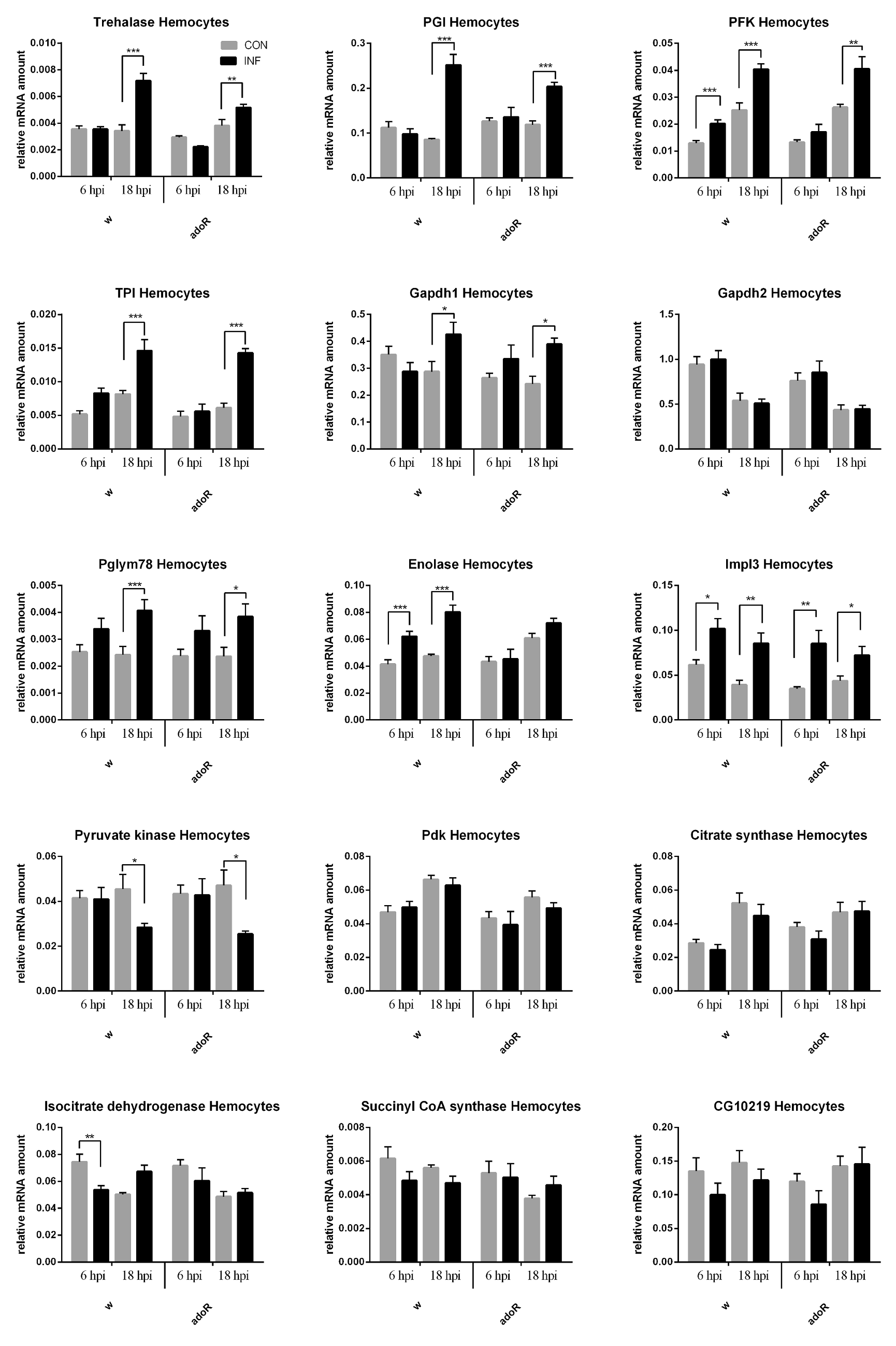

Supplement: S4 Fig — Infection-induced difference in gene expression was analyzed in w and adoR, 6 and 18 hpi. Uninfected individuals are represented by grey columns (CON), infected individuals by black columns (INF). Graphs show mean values relative to Rp49 ± SEM of three independent experiments. Asterisks show statistical significance (*<0.05; **<0.005; ***<0.0005); tested by one-way ANOVA. Gene symbols and the corresponding genes can be found in S1 Table. (TIF) [file pbio.1002135.s005.tif]

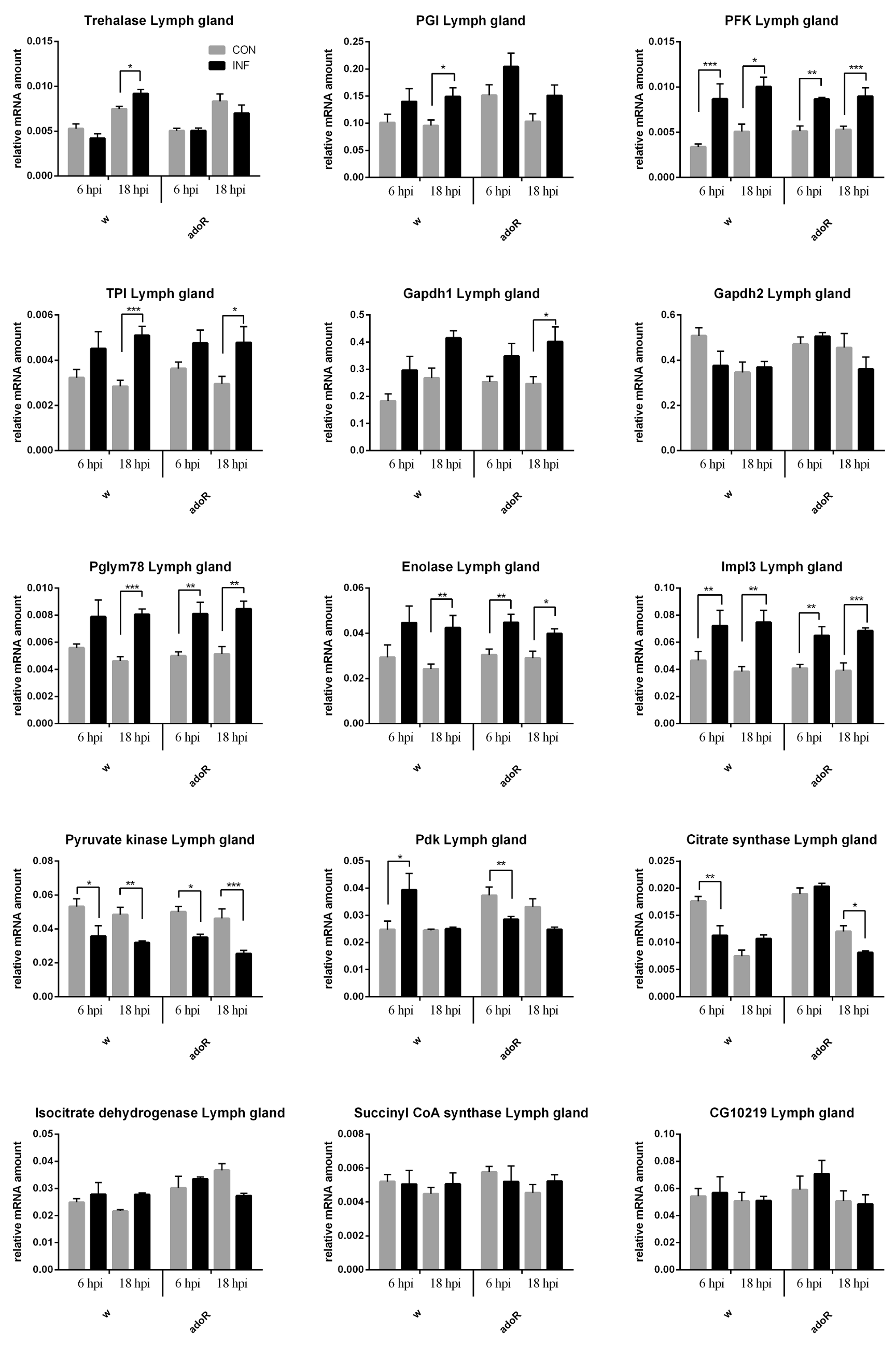

Supplement: S5 Fig — Infection-induced difference in gene expression was analyzed in w and adoR, 6 and 18 hpi. Uninfected individuals are represented by grey columns (CON), infected individuals by black columns (INF). Graphs show mean values relative to Rp49 ± SEM of three independent experiments. Asterisks show statistical significance (*<0.05; **<0.005; ***<0.0005); tested by one-way ANOVA. Gene symbols and the corresponding genes can be found in S1 Table. (TIF) [file pbio.1002135.s006.tif]

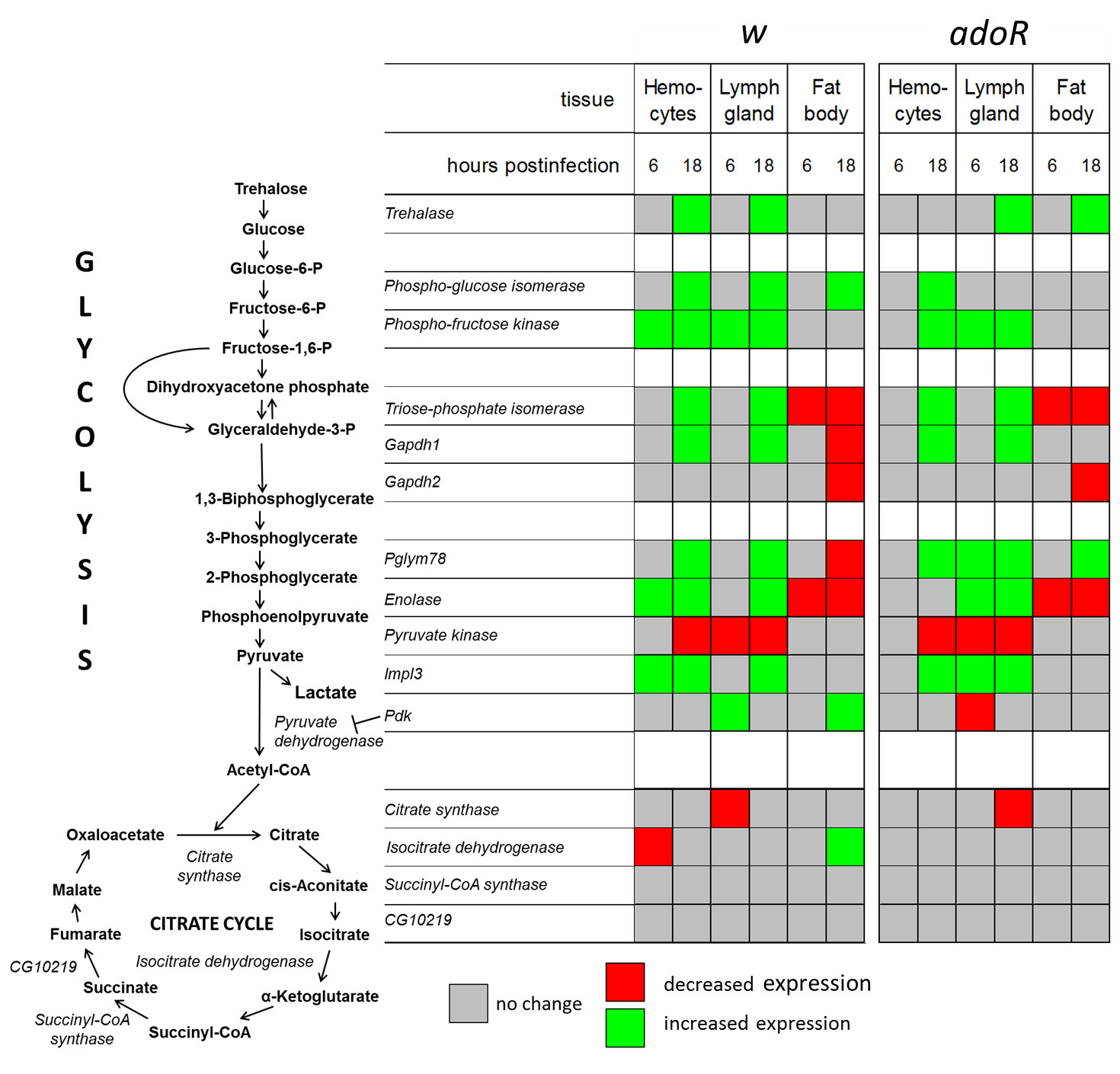

Supplement: S6 Fig — Comparison of infection-induced differences in gene expression between w and adoR in three different tissues (hemocytes, lymph gland, and fat body) and two different time points postinfection (6 and 18 hpi). Green squares—increased expression, red squares—decreased expression, grey squares—no significant difference, white squares—not analyzed. Level of significance p < 0.05; one-way ANOVA. (TIF) [file pbio.1002135.s007.tif]

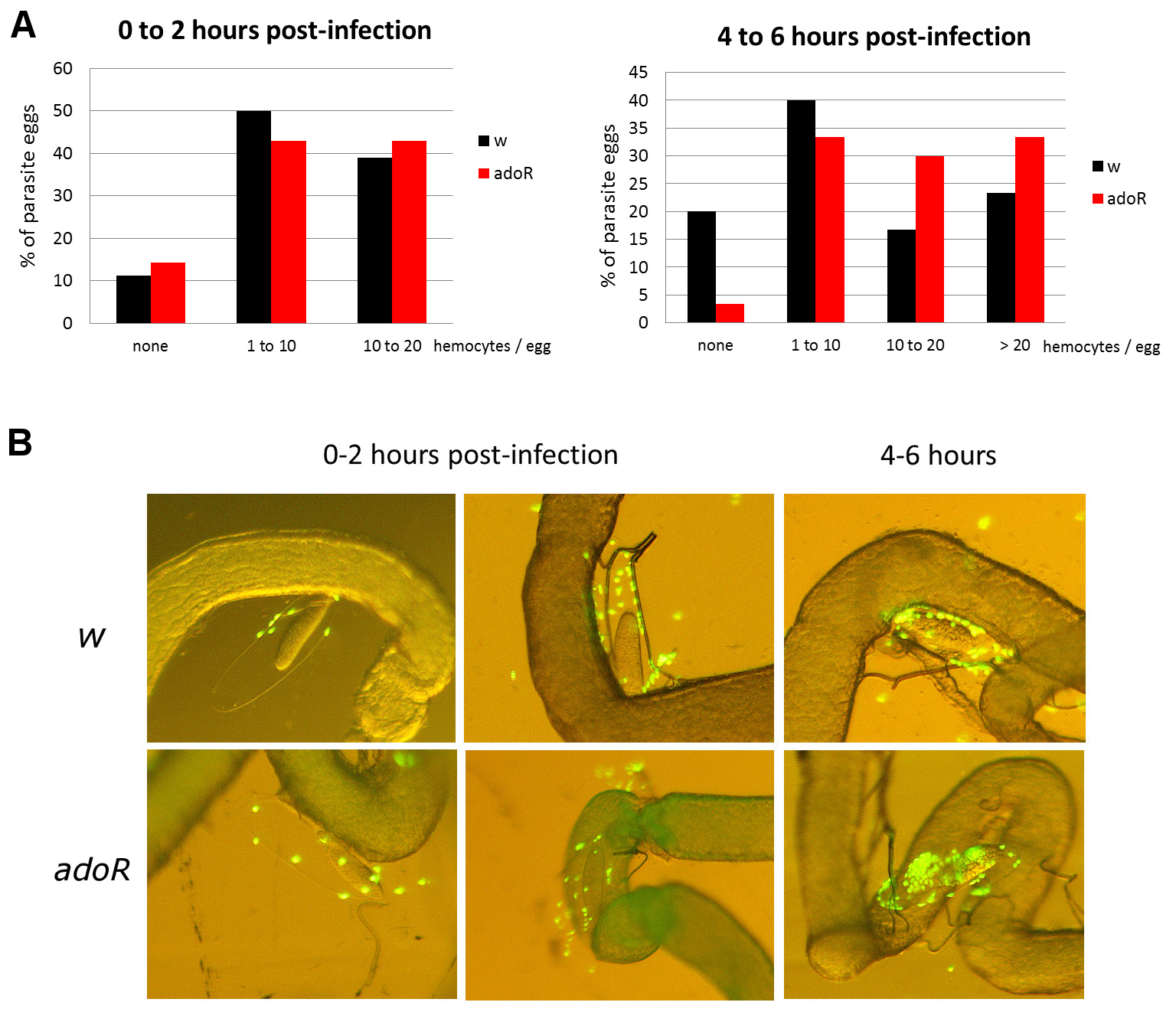

Supplement: S7 Fig — (A) Percentage of eggs with certain number of plasmatocytes (none, 1–10, 10–20, or >20) attached to their surface within the first 2 hpi and 4–6 hpi in w and adoR mutant larvae. (B) Examples of attached Hml>GFP-labeled hemocytes (green fluorescence) to parasitoid wasp egg within the first 2 hpi and 4–6 hpi in w and adoR mutant larvae. (TIF) [file pbio.1002135.s008.tif]

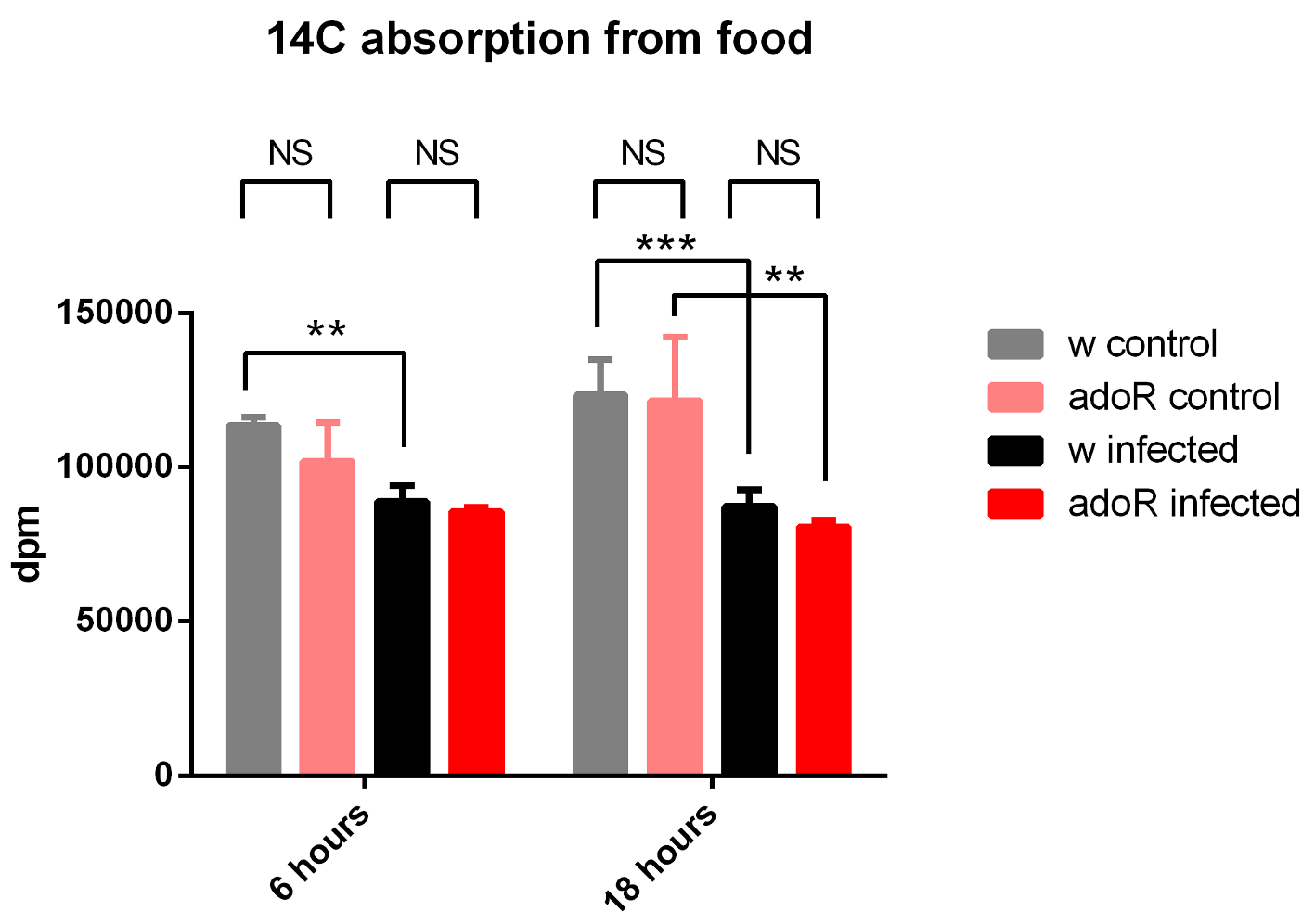

Supplement: S8 Fig — Larvae were fed 14C-glucose (in blue dye-labeled diet) for 20 min and then transferred to normal diet for 5 h in which they absorbed 14C-glucose and cleared their guts. They were then homogenized to analyze how much 14C-glucose they absorbed. There is no difference in absorption between control w and control adoR or infected w and infected adoR both at 6 and 18 hpi (labeled NS for not significant). Interestingly, infected larvae (both w and adoR) absorbed less 14C than control larvae indicating anorexia upon infection. Graph shows uninfected w (grey columns), infected w (black columns), uninfected adoR (pink columns), and infected adoR (red columns) mean values of disintegration of 14C per minute (dpm) per sample ± SEM of three independent experiments, tested by one-way ANOVA. Asterisks show statistical significance (*<0.05; **<0.005; ***<0.0005). (TIF) [file pbio.1002135.s009.tif]

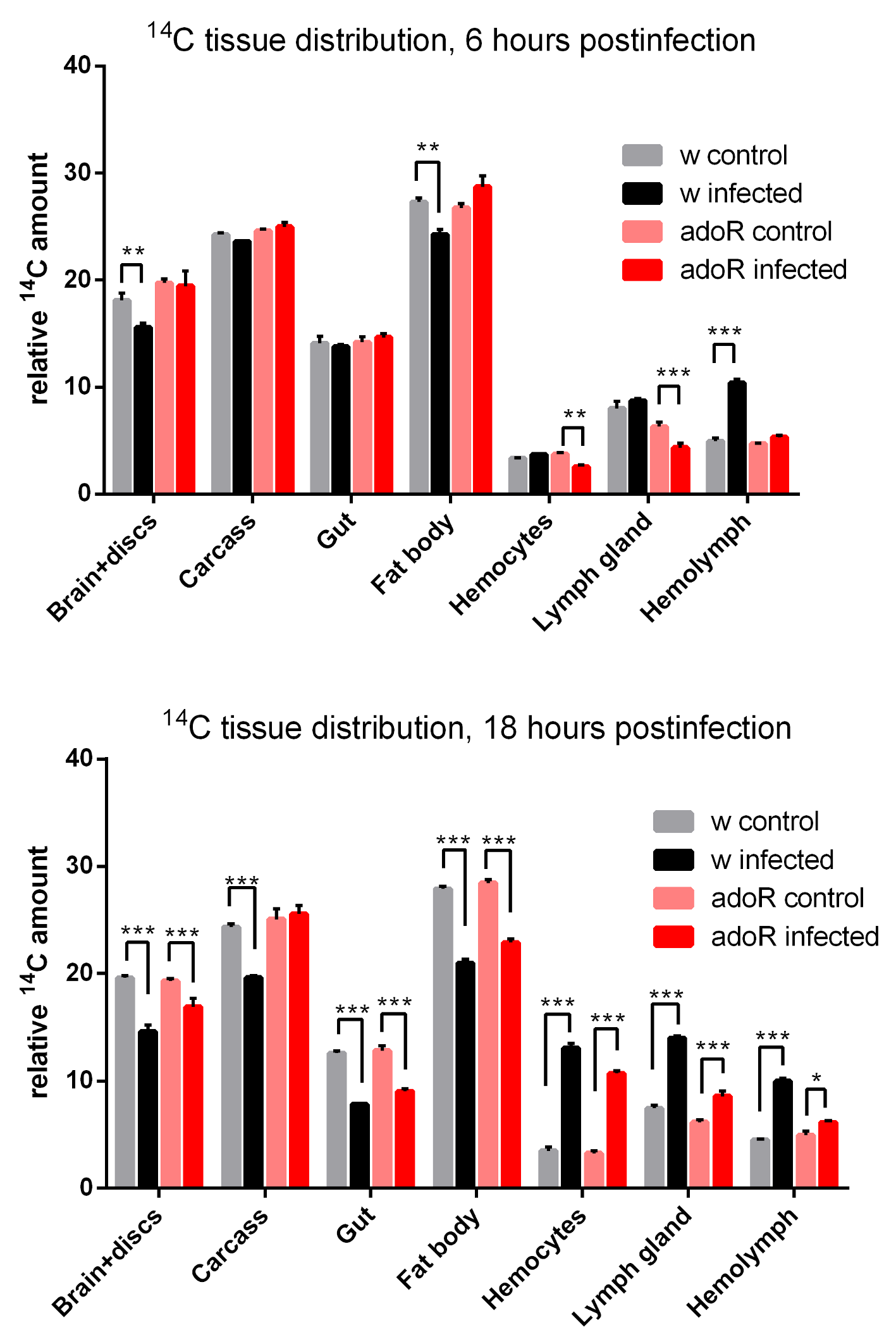

Supplement: S9 Fig — This figure serves as an alternative for Fig 5B to visualize statistical significance. Compared values: uninfected w (grey columns) with infected w (black columns) and uninfected adoR (pink columns) with infected adoR (red columns). Graph shows mean values ± SEM of three independent experiments. Tested by one-way ANOVA with Arc-Sin transformation. Asterisks show statistical significance (*<0.05; **<0.005; ***<0.0005). (TIF) [file pbio.1002135.s010.tif]

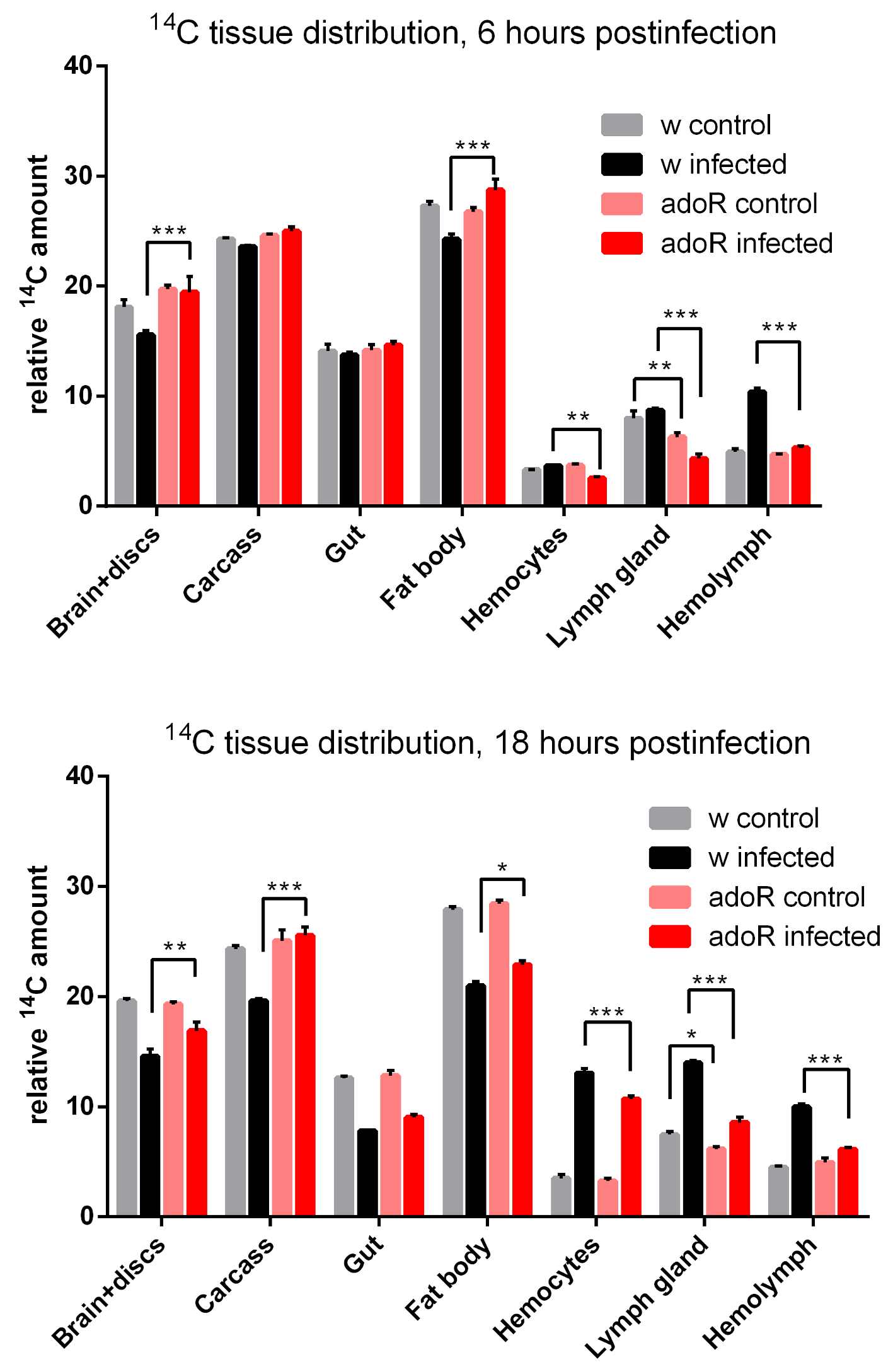

Supplement: S10 Fig — This figure serves as an alternative for Fig 5B to visualize statistical significance. Compared values: uninfected w (grey columns) with uninfected adoR (pink columns) and infected w (black columns) with infected adoR (red columns). Graph shows mean values ± SEM of three independent experiments. Tested by one-way ANOVA with Arc-Sin transformation. Asterisks show statistical significance (*<0.05; **<0.005; ***<0.0005). (TIF) [file pbio.1002135.s011.tif]

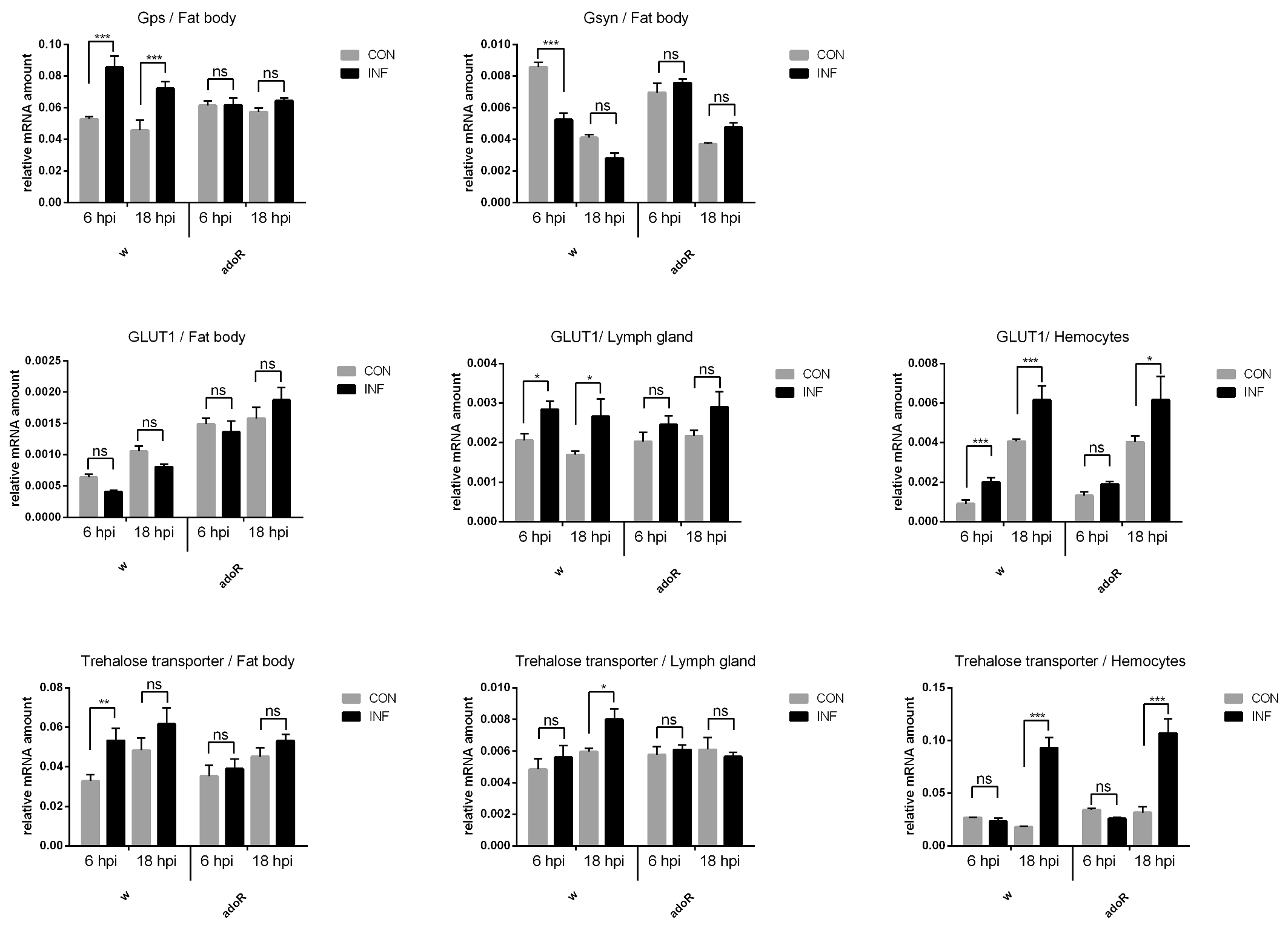

Supplement: S11 Fig — Graphs display infection-induced differences in expression level of Trehalose transporter TreT1-1, Glucose transporter 1 (Glut1), Glycogen synthase (Gsyn), and Glycogen phosphorylase (Gps) in fat body, lymph gland, and circulating hemocytes at 6 and 18 hpi. Uninfected individuals marked as CON (grey columns), infected individuals marked as INF (black columns). Graph shows mean values ±SEM of three independent experiments. Asterisks show statistical significance (*<0.05; **<0.005; ***<0.0005; ns for nonsignificant difference); tested by one-way ANOVA. (TIF) [file pbio.1002135.s012.tif]

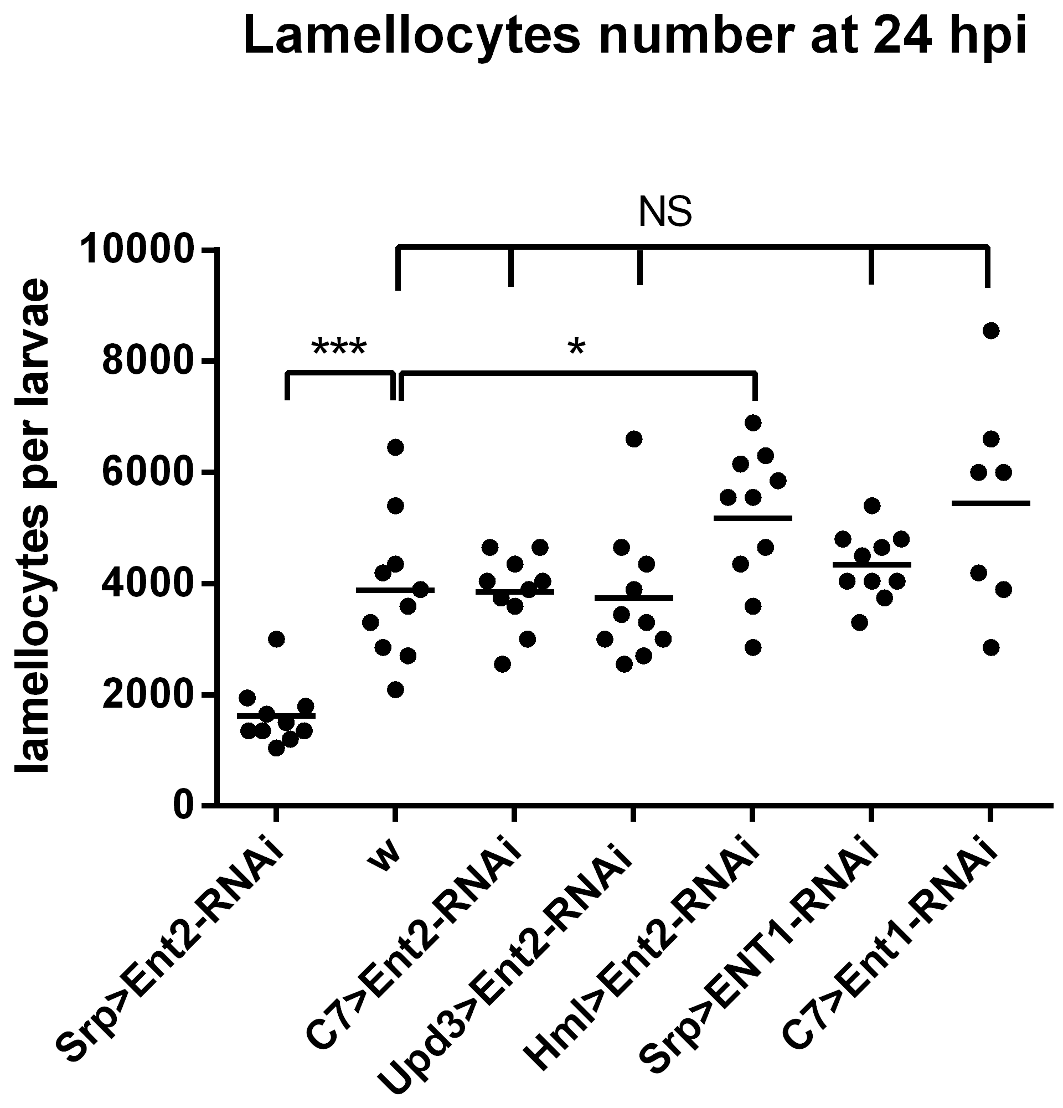

Supplement: S12 Fig — RNAi was induced by driving UAS-Ent1-RNAi (VDRC ID 109885) and UAS-Ent2-RNAi (VDRC ID 100464) by various Gal4 drivers: Srp-Gal4 expressed in all cells of hematopoietic lineage and fat body, Upd3-Gal4 and Hml-Gal4 in differentiated hemocytes and C7-Gal4 in fat body. Lamellocytes were counted based on morphology using DIC at 24 hpi. Only combination of Srp>Ent2-RNAi significantly decreased lamellocytes. Results were tested by one-way ANOVA, each point in graph represents number of lamellocytes in one individual larva. Asterisks show statistical significance (*<0.05; **<0.005; ***<0.0005; NS for nonsignificant difference). (TIF) [file pbio.1002135.s013.tif]

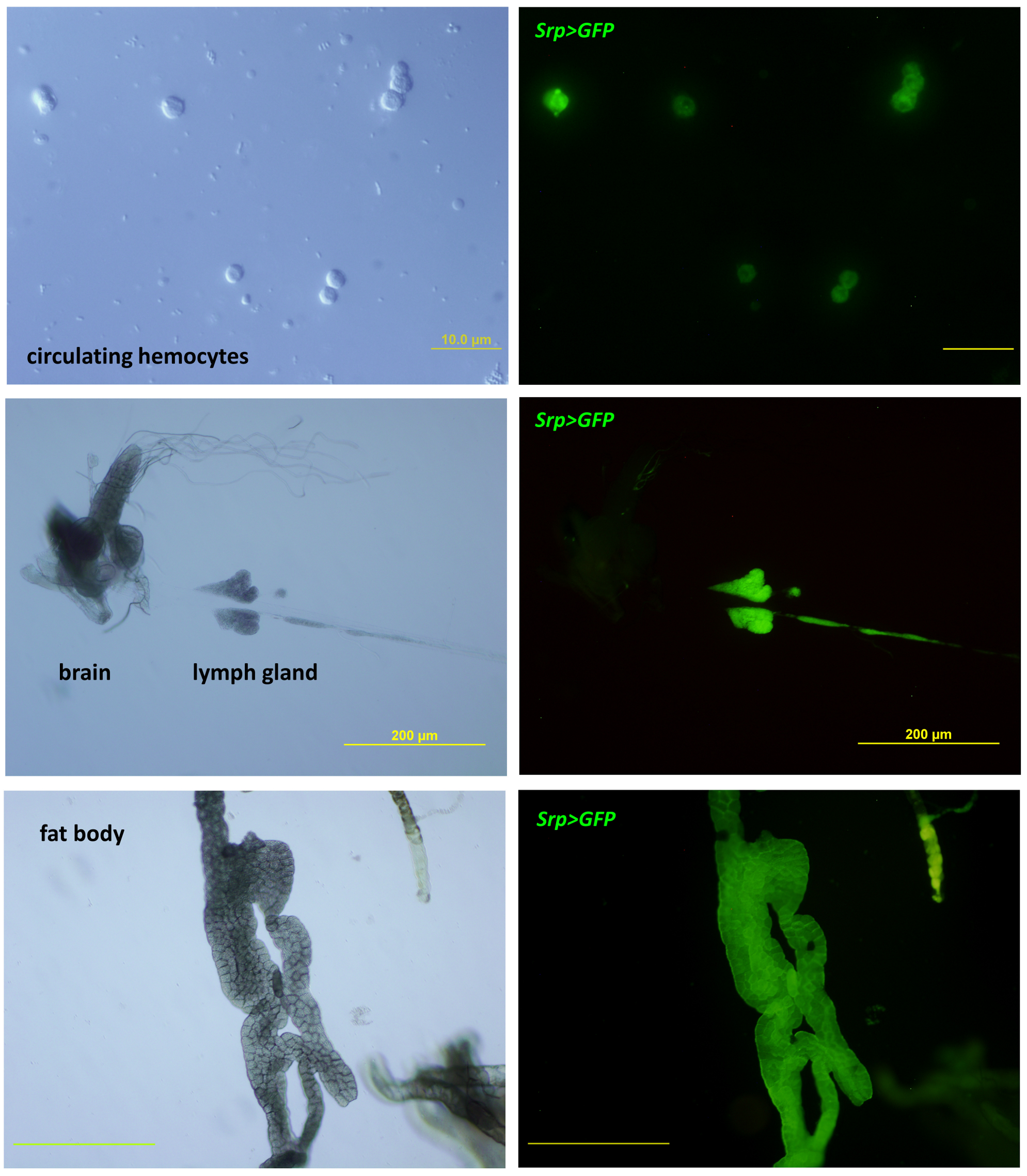

Supplement: S13 Fig — Srp-Gal4 driver expression was visualized by crossing to UAS-GFP. Strong expression was detected in all cells of hematopoietic lineage as demonstrated by the GFP fluorescence in circulating hemocytes and all cells in the lymph gland. The expression was also detected in fat body but it was undetectable in the brain besides weak expression in nerve cords. Left panels show DIC image corresponding to GFP fluorescence images on right. (TIF) [file pbio.1002135.s014.tif]

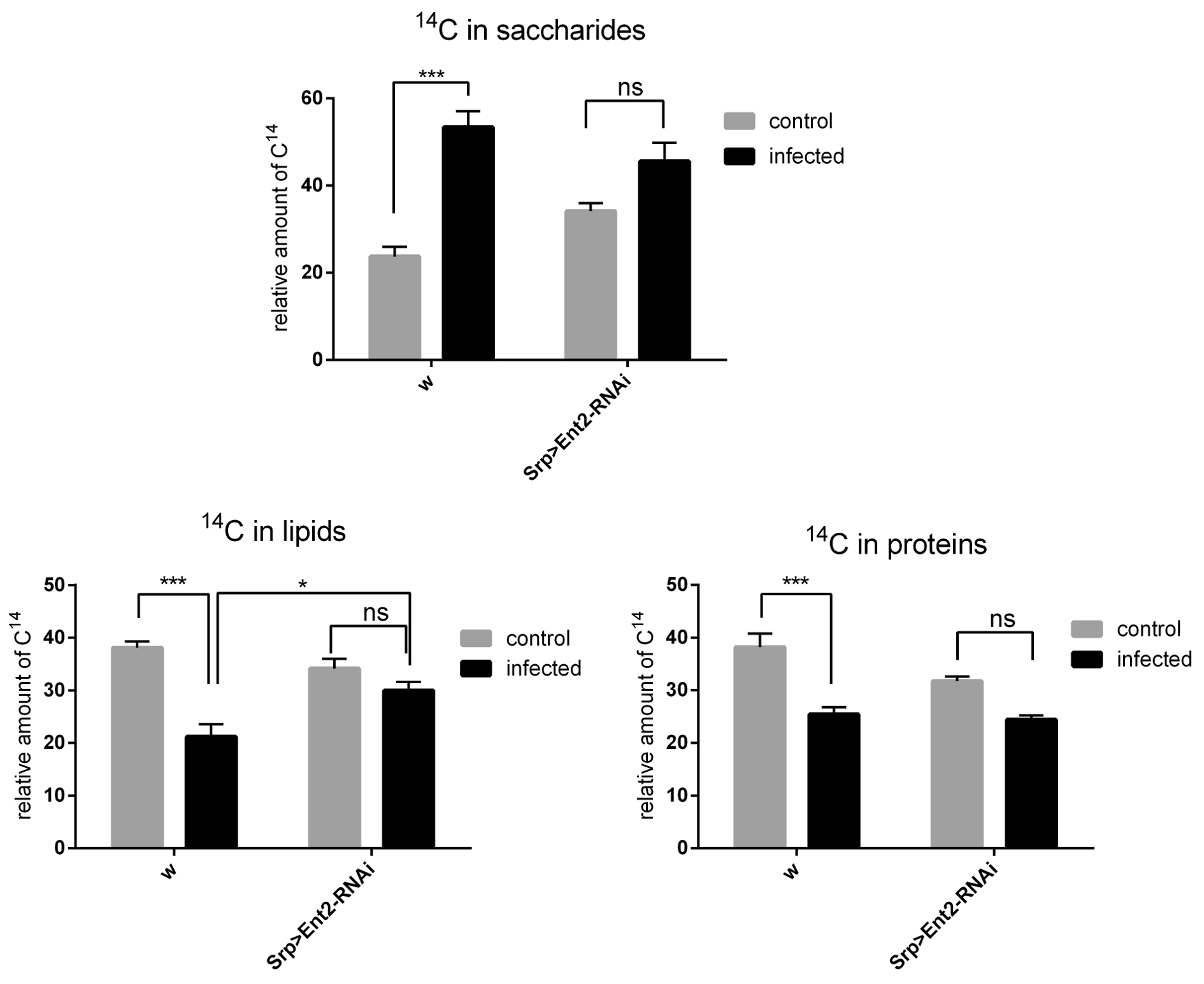

Supplement: S14 Fig — Comparison of incorporation of 14C into macromolecules (saccharides, lipids, and proteins) tested by two-way ANOVA. Uninfected individuals are marked as control (grey columns), infected individuals are marked as infected (black columns). Graphs show mean values ± SEM of three independent experiments. Asterisks show statistical significance (*<0.05; **<0.005; ***<0.0005; ns for not significant). (TIF) [file pbio.1002135.s015.tif]

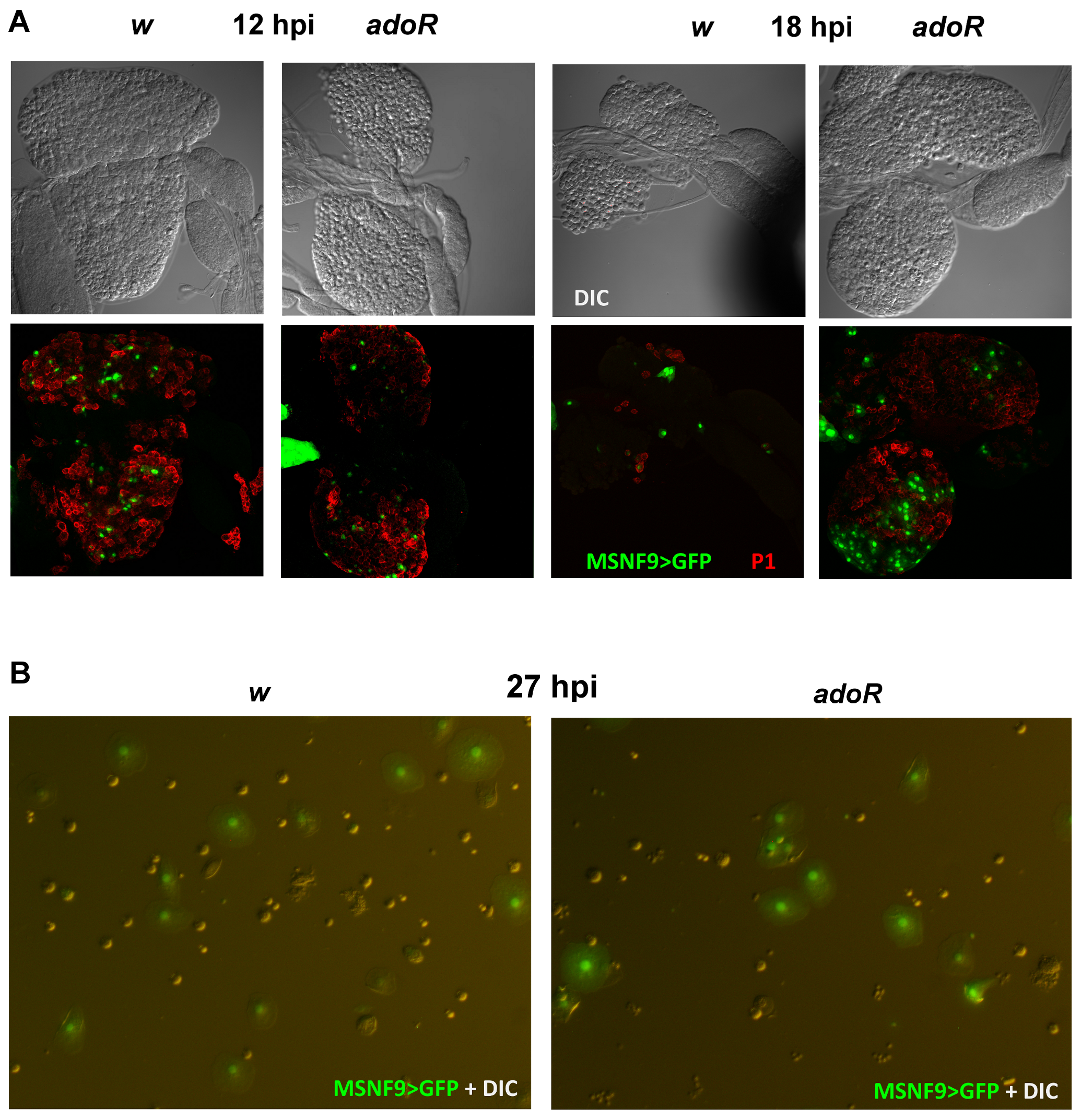

Supplement: S16 Fig — (A) Expression of MSNF9>GFP (green) together with plasmatocytes-specific P1 marker (red) in the lymph gland upon infection at 12 and 18 hpi. Both w and adoR express the lamellocyte marker indicating that adoR is able to differentiate lamellocytes but there is usually less MSNF9>GFP signal in adoR at 12 hpi. While w sometimes already releases lamellocytes into circulation at 18 hpi (as demonstrated by disintegrated lymph gland in DIC picture), adoR has lymph gland still compact at 18 hpi, but with increasing number of MSNF9>GFP positive cells further demonstrating ability of adoR to differentiate lamellocytes but with lower speed. Top—DIC, bottom—fluorescence confocal image. (B) MSNF9>GFP positive lamellocytes in circulation at 27 hpi are present in both w and adoR and their morphology is indistinguishable. Fluorescence and DIC-combined micrographs. (TIF) [file pbio.1002135.s017.tif]

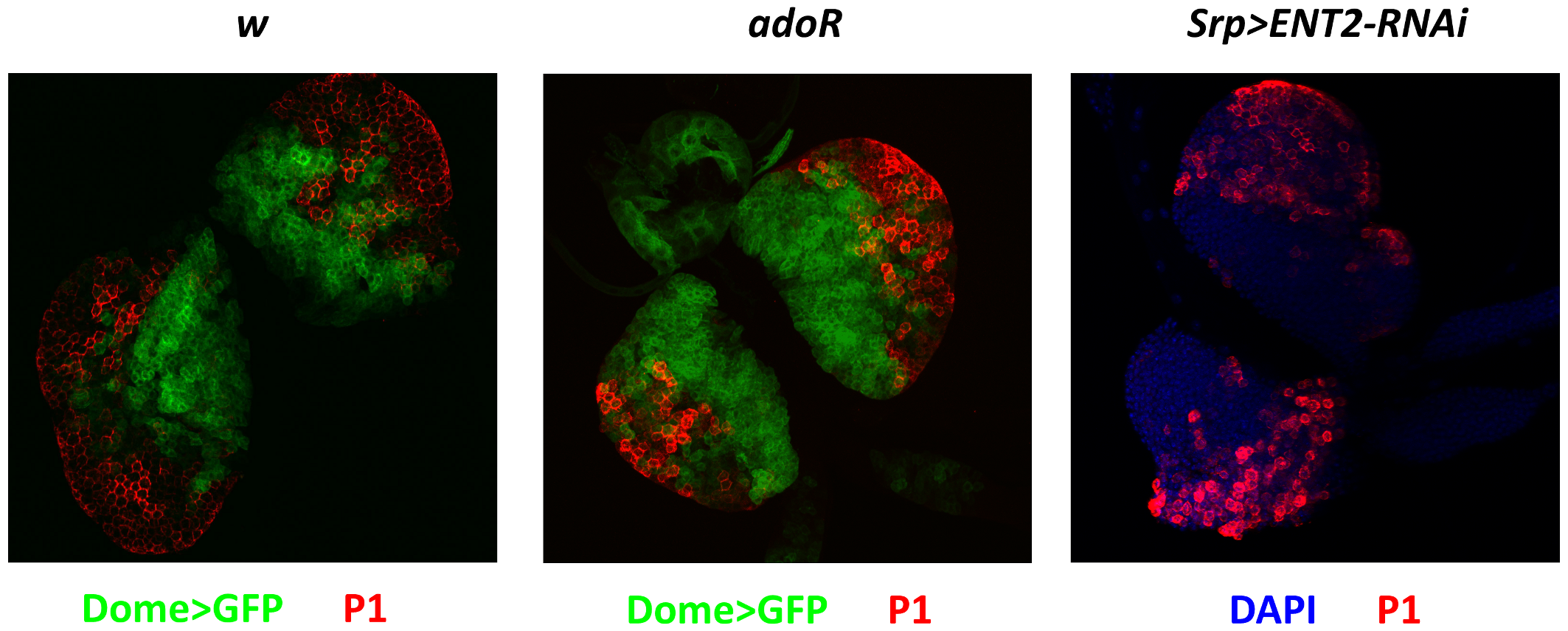

Supplement: S17 Fig — Morphology and zonation of the lymph gland in 72-h-old larvae (corresponding to time of infection) was checked by expression of medullary zone-specific Dome>GFP marker (prohemocyte containing zone; green) and differentiated plasmatocyte-specific P1 marker for cortical zone (red) in w and adoR. Only P1 marker was used in Srp>ENT2-RNAi and DAPI (nuclear staining) for overall morphology to define medullary zone by the absence of P1. In all three genotypes, the zonation and morphology was comparable for multiple samples indicating that there is no gross effect of the used genetic manipulations on the lymph gland development prior to infection. (TIF) [file pbio.1002135.s018.tif]
