# Supplementary material for: Extracellular Adenosine Mediates a Systemic Metabolic Switch during Immune Response
Source: PLoS Biol. 2015 Apr 27;13(4):e1002135. doi: 10.1371/journal.pbio.1002135 (PMC4411001; doi:10.1371/journal.pbio.1002135)
Supplement: S1 Text — (DOCX) [file pbio.1002135.s020.docx]

**S1 TEXT: EXTENDED MATERIALS AND METHODS**

SUPPORTING INFORMATION for: Extracellular adenosine mediates a systemic metabolic switch during immune response - Adam Bajgar, Katerina Kucerova, Lucie Jonatova, Ales Tomcala, Ivana Schneedorferova, Jan Okrouhlik, Tomas Dolezal

**Fly stocks**

All strains were backcrossed at least 10 times to *w^1118^* genetic background; *w^1118^* was used as a control in all experiments. *adoR* mutant was homozygous for *adoR^1^* mutation (FBal0191589) [1]. RNAi lines originated from Vienna Drosophila Rnai Center (VDRC): *UAS-Ent1-RNAi* (ID 109885) and *UAS-Ent2-RNAi* (ID 100464). *SrpD-Gal4* [2], *Upd3-Gal4* [3], *MSNF9-GFP* [4] and *Dome-Gal4 UAS-GFP* were obtained from Michele Crozatier, *HmlΔ-Gal4* from Bruno Lemaitre and *C7-Gal4* from Marek Jindra.

**Fly and wasp culture**

Flies were grown on cornmeal medium (8% cornmeal, 5% glucose, 4% yeast, 1% agar, 0.16% methylparaben) at 25°C. Parent flies were allowed to lay eggs on 60-mm petri dishes with the cornmeal medium in small cages (Flystuff) for 1-2 hours and progeny larvae were collected to fresh media at 72 hours after egg laying (AEL). For dietary treatment, larvae were transferred upon infection to 12%-glucose medium (8% cornmeal, 12% glucose, 4% yeast, 1% agar, 0.16% methylparaben). Parasitoid wasps *Leptopilina boulardi* were kept on sugar agar media (6% sucrose, 1.5% agar, 0.75% methylparaben) and grown by infection of wild-type Drosophila larvae.

**Parasitoid wasp infection**

Early 3^rd^ instar larvae (all genotypes around 72 h AEL) were infected by parasitoid wasp *Leptopilina boulardi* - this represents T0 (0 hours post infection or 0 hpi) in all presented data (including uninfected control). Weak infection (1-2 eggs per larva) was used for resistance and pupation analysis since the stronger infection almost always lead to host death. Strong infection (4-7 eggs per larva) was used in all other cases since it ensured a robust and more uniform immune response; the developmental delay compared to uninfected animals was increased from 7 h (weak infection) to 18 h (strong infection) but still majority of larvae (>80%) pupated demonstrating that even strong infection allowed to proceed in development. Infections were always verified by a dissection of representative sample of larvae (5-10%; at least 10 larvae) and infections were used only when 90% or more of the larvae contained the desired number of parasitic eggs. Infections were performed on 60 mm-petri dishes with cornmeal medium for 10 min with periodic disruption of infecting wasps for weak infection and for 90 min for strong infection with ratio of 2 larvae per 1 female wasp.

**Resistance and pupation analysis**

To determine pupation rate and resistance to parasitoid, infected/control larvae were placed into fresh vials (1 experiment=30 larvae/vial, 3 vials/genotype; 4 independent experiments). Pupation rate was determined by counting newly appeared pupae every 6 h and incremental percentage of number of pupae per total number of infected/control larvae in particular time point post infection was plotted; Log-rank survival analysis was used for comparison. For resistance, we first dissected 20 larvae per experiment from each genotype to count fully melanized wasp egg (winning host) or surviving wasp larva (winning parasitoid). Second, we counted all emerged adult flies as surviving the infection and flies without any egg (i.e. uninfected individuals) were excluded from the total number in the experiment. Adult wasps emerged from the vial were counted as adult parasitoid winners.

**Hemocyte counting**

Hemocytes were obtained from larvae by cuticle tearing in PBS (15 µl per larva) and counted based on morphology (DIC) and by presence of MSNF9-GFP marker for lamellocytes in Neubauer hemocytometer (Brand GMBH).

**Gene expression analysis**

RNA was isolated by Trizol reagent (Ambion) according to manufacturer protocol. DNA contamination was removed by using Turbo DNAse free kit (Ambion) according to the protocol (37°C 30 min) with subsequent inactivation of DNAse by DNAse inactivation reagent (5 min at RT, spin 13000 RPM at RT). Reverse transcription was done by Superscript SSIII reverse transcriptase (Invitrogen) and amounts of mRNA of particular genes were quantified by IQ sybr green supermix mastermix (BioRAd) on CFX 1000 Touch Real time cycler (BioRad). In all cases, the expression was normalized to expression of Ribosomal protein 49 (Rp49) and values relative to Rp49 amounts were compared and shown in graphs. Primers are listed in S1 Table.

**^14^C-glucose distribution into tissues and macromolecules**

Relative distribution of dietary glucose into different tissues was determined by tracing the ^14^C from U-^14^C-glucose. Infected and control larvae were fed either 73 h AEL or 91 h AEL for 20 min on diet containing radioactively labeled ^14^C-glucose – 100 µl of D[U-^14^C] glucose (10.6 Gbq/nmol; Amersham Biosciences) were added to 15 ml of heat inactivated yeast paste with 5% regular glucose. Larvae were then transferred to normal diet for 5 h to ingest and metabolize the ^14^C-glucose. After 5 h (corresponding to either 6 hpi or 18 hpi; see S1 Fig. for detailed time scales), larvae were dissected and their tissues stored at -20°C until ^14^C quantification (see further). Each sample contained pooled tissues from 30 larvae that were dissected as follows: larvae were ripped in PBS and all hemolymph was collected, centrifuged to pellet hemocytes and divided to hemocyte and hemolymph fractions. Brains with attached discs and wing discs, whole guts, whole fat bodies and lymph glands were separated by dissection in PBS and the remnants were used as carcass. All tissues were hydrolyzed overnight by Solvable (Packard Bioscience) prior to scintillation measurement.

Relative ^14^C distribution into macromolecules was determined as follows: larvae were infected and fed on radioactively labeled glucose as mentioned above and 30 larvae were homogenized 5 h post-feeding in 500 µl of PBS. 100 µl were used for a direct measurement of total absorbed ^14^C (100 µl of Homogenate + 400 µl of Solvable), 200 µl were used for separation of lipids and saccharides according to Bligh and Dyer protocol [5] and 200 µl were used for separation of proteins by their precipitation with trichloracetic acid. All samples were in the last step mixed with 200 µl Solvable (Packard Bioscience) and mixed with scintillation cocktail (UltimaGold, Perkin Elmer) and number of disintegrations of ^14^C per minute was measured on liquid scintillation analyzer (TriCarb 2900TR, Packard Bioscience).

Preparation of sample for absorption of total ^14^C

100 µl of homogenate from 30 larvae

400 µl of Solvable (Packard Bioscience)

Overnight hydrolysis

1000 µl of scintillation cocktail

Separation of lipid fraction

200 µl of homogenate from 30 larvae

750 µl of chlorophorm : methanol (2:1); vortex

250 µl of chlorophorm; vortex

250 µl of H_2_O; vortex

Centrifuge 100 RPM 5 min at RT

Separate lower (chlorophorm) phase, let evaporate

Separation of saccharide fraction

Use 400 µl of upper phase from previous lipid fraction procedure

100 µl of trichloracetic acid to precipitate all proteins

Incubate 20 min on ice

Centrifuge 14000 RPM 10 min at 4°C

Transform supernatant into fresh eppendorf tube

Separation of proteins

200 µl of homogenate from 30 larvae

50 µl of trichloracetic acid to precipitate all proteins

20 min on ice

Centrifuge 14000 RPM 10 min at 4°C

200 µl of ice cold acetone

Centrifuge 14000 RPM 5 min at 4°C

Hydrolyze pelleted proteins in 200 µl of Solvable (overnight)

**Metabolites measurement**

Circulating glucose and trehalose, tissue trehalose and glycogen were measured by approaches published in [6]. Briefly, hemolymph was collected by tearing cuticle of approx. 50 larvae on ice, hemocytes were pelleted by centrifugation (800xg, 5 min, 4°C) and 4 µl of supernatant were used for protein measurement and 4 µl were denatured by heating 75°C for 10 min and stored at -80°C. Tissue samples were homogenized in 1xPBST (PBS-0,03% Tween), large fragments were pelleted by centrifugation (800xg, 5 min, 4°C), half of the sample was used for protein quantification and the rest was denatured by heating 75°C for 10 min and stored in -80°C. Glucose was determined by GAGO-20 kit (Sigma) according to supplier protocol using spectrophotometric measurement at 540 nm. Trehalose samples were first treated by trehalase enzyme mix (Sigma T8778) overnight at 37°C and glycogen samples with amyloglucosidase enzyme (Fluka-Sigma 10115) for 30 min. Protein concentration was analyzed by using Bradford measurement. Samples were homogenized and proteins were solved in 1xPBS. 100 µl of protein sample were mixed with 10 µl of Bradford solution (10 mg of Brilliant blue, 5 ml of 96% Ethanol, 10 ml of 85% Phosphoric acid in 100 ml of solution). Concentration of proteins was derived from absorbance of reaction solution at 595 nm.

Lipids from dissected fat bodies (5 animals/sample) were extracted with chloroform: methanol (2:1) solution following the method of [7] as modified by [8]. Each sample was homogenized in the solution, extracted and 100 µl were used for the lipid determination. The procedure employed the ion trap LTQ mass spectrometer coupled to Surveyor HPLC system equipped with Accela autosampler with the thermostat chamber (all by Thermo, San Jose, CA, USA). The samples (5 µl) were injected and separated using the Gemini column 250 x 2 mm i.d. 3 µm (Phenomenex, Torrance, CA, USA). The mobile phase consisted of (A) 5 mM ammonium acetate in methanol, (B) water, and (C) 2-propanol. The mass spectrometer was operated in the positive and negative ion detection mode at + and -4kV with capillary temperature at 220°C. The nitrogen was employed as shielding and auxiliary gas. Mass range of 140-1400 Da was scanned every 0.5 s to obtain the full scan ESI mass spectra of lipids. For the investigation of the lipid molecules structures, the collisionally induced decomposition multi-stage ion trap tandem mass spectra MS^2^ in both polarity settings were simultaneously recorded with a 3 Da isolation window. Peak areas of detected lipids were used for estimation of their relative content in the analysed samples.

**Imaginal disc size measurement**

Wing discs were dissected from infected and control larvae at 90 h AEL (18 hpi), discs were documented using inverted microscope Olympus IX71 with Olympus DP70 camera. Size was determined by FIJI software.

**Immunohistochemistry**

Lymph glands were dissected from 72 hours-old larvae or at specific times post infection, fixed in paraformaldehyde and stained according to [9]. To visualize plasmatocytes, mouse P1 antibody (1:30) was used in combination with anti-mouse Alexa Fluor R 647 goat anti-mouse IgG (H+L) (Molecular Porbes), GFP fluorescence was used with *Dome>GFP* marker for medullary zone and *MSNF9>GFP* marker for lamellocytes. DAPI was used to stain nuclei or DIC contrast to visualize the overall morphology of the lymph gland. Confocal microscopy (Olympus U-TB 190) was used for documentation.

**Data analysis and statistics**

All data were analyzed and graphed by GraphPad Prism 6 (GraphPad Software, Inc.)

**REFERENCES**

1. Dolezal T, Dolezelova E, Zurovec M, Bryant PJ. A role for adenosine deaminase in Drosophila larval development. PLoS Biol. 2005;3: e201. doi:10.1371/journal.pbio.0030201

2. Crozatier M, Ubeda J-M, Vincent A, Meister M. Cellular immune response to parasitization in Drosophila requires the EBF orthologue collier. PLoS Biol. 2004;2: E196–E196. doi:10.1371/journal.pbio.0020196

3. Agaisse H, Petersen U-M, Boutros M, Mathey-Prevot B, Perrimon N. Signaling Role of Hemocytes in Drosophila JAK/STAT-Dependent Response to Septic Injury. Dev Cell. 2003;5: 441–450. doi:10.1016/S1534-5807(03)00244-2

4. Tokusumi T, Sorrentino RP, Russell M, Ferrarese R, Govind S, Schulz R a. Characterization of a lamellocyte transcriptional enhancer located within the misshapen gene of Drosophila melanogaster. PloS One. 2009;4: e6429. doi:10.1371/journal.pone.0006429

5. Bligh EG, Dyer WJ. A rapid method of total lipid extraction and purification. Can J Biochem Physiol. 1959;37: 911–917.

6. Tennessen JM, Barry W, Cox J, Thummel CS. Methods for studying metabolism in Drosophila. Methods. 2014;68: 105–115. doi:10.1016/j.ymeth.2014.02.034

7. Folch J, Lees M, Stanley GHS. A Simple Method for the Isolation and Purification of Total Lipides from Animal Tissues. J Biol Chem. 1957;226: 497–509.

8. Kostal V, Simek P. Changes in fatty acid composition of phospholipids and triacylglycerols after cold-acclimation of an aestivating insect prepupa. J Comp Physiol B. 1998;168: 453–460. doi:10.1007/s003600050165

9. Crozatier M, Vincent A. Requirement for the Drosophila COE transcription factor Collier in formation of an embryonic muscle: transcriptional response to notch signalling. Development. 1999;126: 1495–1504.
